# Adherence to the EAT-Lancet Planetary Health Diet in Portugal and its associations with socioeconomic and lifestyle factors

**DOI:** 10.1007/s00394-025-03661-6

**Published:** 2025-04-09

**Authors:** Catarina Carvalho, Daniela Correia, Carla Lopes, Duarte Torres

**Affiliations:** 1 EPIUnit – Instituto de Saúde Pública, Universidade do Porto, Rua das Taipas, nº 135, 4050 - 600 Porto, Portugal; 2 Laboratório para a Investigação Integrativa e Translacional em Saúde Populacional (ITR), Porto, Portugal; 3 Faculdade de Ciências da Nutrição e Alimentação, Universidade do Porto, Porto, Portugal; 4 Departamento de Ciências da Saúde Pública e Forenses e Educação Médica, Faculdade de Medicina, Universidade do Porto, Porto, Portugal

**Keywords:** EAT-Lancet Planetary Health Diet, Diet quality, Dietary sustainability, Dietary survey

## Abstract

**Purpose:**

The Planetary Health Diet (PHDiet) proposed by the EAT-Lancet Commission is expected to bear health and environmental benefits. This study assesses adherence to the PHDiet in Portuguese adults and its associations with socioeconomic and lifestyle factors. For that, an adapted PHDiet score was computed, and its construct validated.

**Methods:**

Data from the National Food and Physical Activity Survey 2015–2016 were used for this analysis, covering a representative sample of 3852 adults with two non-consecutive dietary interviews (8–15 days apart). Adherence to the PHDiet was measured through a score (ranging from 0 to 140), afterwards divided into terciles (T1–T3). Multinomial regression models were used (i) to assess the construct validity through associations with diet quality based on WHO recommendations and diet environmental impact using greenhouse gas emissions (GHGE) and land use (LU); (ii) to investigate associations between adherence to the PHDiet and socioeconomic and lifestyle characteristics. The prevalence of consumption of PHDiet components above/below the recommendations was estimated.

**Results:**

Adherence to the PHDiet was generally low (36.0, 95% CI 35.4–36.6), with high consumption of meat and added sugars and low consumption of pulses, nuts, and whole grains. Higher PHDiet scores were found for diets with lower environmental impact (GHGE: OR_T1vsT3_:1.31; 95% CI 1.26; 1.37; LU: OR_T1vsT3_:1.25; 95% CI 1.21; 1.29), lower animal protein intake levels (OR_T1vsT3_:1.11; 95% CI 1.06; 1.16) and higher diet quality (OR_T1vsT3_:0.70; 95% CI 0.68; 0.72), verifying the construct validity. Men (OR_T1vsT3_:1.32; 95% CI 1.12; 1.55), intermediate-educated individuals (OR_T1vsT3_:1.43; 95% CI 1.16; 1.75), and those facing food insecurity (OR_T1vsT3_:1.79; 95% CI 1.36; 2.38) had higher odds of having lower scores.

**Conclusion:**

Low adherence to the PHDiet is associated with several socioeconomic and lifestyle factors. This highlights the need to implement targeted public health policies that encourage shifts towards a healthier and more sustainable dietary pattern.

**Supplementary Information:**

The online version contains supplementary material available at 10.1007/s00394-025-03661-6.

## Introduction

Dietary intake is intrinsically connected with human health and environmental sustainability [1, 2]. According to the Global Burden of Disease Study 2019, dietary risk factors are the fifth and third-leading Level- 2 risk factors for attributable Disability-Adjusted Life Years (DALYs) and premature deaths worldwide [3]. Moreover, the food system is one of the most significant contributors to climate change, as measured by greenhouse gas (GHG) emissions [4]. In response to this issue, in 2019, the EAT-Lancet Commission proposed the Planetary Health Diet (PHDiet), a global healthy reference diet, mainly consisting of vegetables, fruits and with whole grains, plant-based protein sources, unsaturated oils and decreased amount of meat, dairy, added sugar and starchy vegetables [5].

The dietary shift for adopting the PHDiet is estimated to lead to a 19% decrease in premature deaths globally [6]. Accordingly, adherence to the PHDiet and its associations with several health effects have been studied in different settings, with promising results, especially related to cardiovascular and metabolic outcomes [7–13]. Considering the environmental domain, a shift towards the PHDiet alone is expected to lead to significant improvements regarding greenhouse gas emissions reduction, with estimates pointing to an overall decrease of 17% foreseen worldwide adoption of the PHDiet [14].

Still, this necessary global-level dietary shift needs to be supported by the development of regional-specific public policies [5]. The design and effectiveness of such policies benefit from knowledge of the current status of that specific context, of the dietary patterns of the target population and the individual, social, cultural, economic, and environmental characteristics that influence these dietary patterns [15].

In Portugal, no data is available on the adherence of the population to the PHDiet referential and on the characteristics of the individuals that mostly associate with its adherence. Data from the National Food and Physical Activity Survey (IAN-AF 2015–2016) [16] show that animal-based foods (including meat, fish, seafood, and eggs) are the second-largest contributor to total energy intake, which falls off the recommendations for healthy and sustainable diets, that favour plant-based foods as the basis for the overall dietary pattern [5]. Moreover, a considerable proportion of individuals present an inadequate intake of fruits, vegetables and pulses. Poor dietary habits can lead to nutrient deficiencies and several adverse health outcomes. Particularly, in this country, dietary risk factors are pointed out as one of the top responsible risk factors for a relevant share of the preventable burden of disease [17]. Regarding the environmental domain, a previous study has estimated that the food system accounts for around one-third of the country’s GHG emissions [4].

All these figures indicate a high potential in the Portuguese population for a dietary shift towards sustainable and healthier dietary patterns. However, not all individuals within a certain population have identical dietary behaviours, and some might require more profound behavioural changes. Notably, food consumption is determined by gender and socio-economic factors, including education, working conditions, family household composition, and other lifestyle characteristics [18, 19]. Thus, understanding and considering these differences when evaluating adherence to a specific dietary pattern is of utmost importance for informing research on the topic and targeted public health policies aimed at improving the dietary habits of a population such as the Portuguese.

Thus, this study aims to evaluate the adherence to the PHDiet in the Portuguese population and explore which socioeconomic and lifestyle-related factors are associated with it. Moreover, this study aims to assess the construct validity of the adapted PHDiet score by evaluating whether higher PHDiet scores are associated with better nutritional health and environmental outcomes, as hypothesised.

## Methods

### Study population and dietary assessment methods: IAN-AF 2015–2016

This study was conducted using data collected under the Portuguese National Dietary Survey, the IAN-AF 2015–2016 [20, 21], a cross-sectional survey evaluating food consumption, anthropometric characteristics, and physical activity habits of a representative sample of the Portuguese population, according to the EU Menu project European guidelines [22]. Below is a brief description of the IAN-AF population and methodologies; further methodological details can be found in previous publications [20, 21].

The participants (n = 5811) of IAN-AF 2015–2016, aged between 3 months and 84 years old, were selected from the National Health Registry by multistage sampling. In the present study, we used a sub-sample of 3852 adults over 18 years old who completed two dietary assessment interviews.

In IAN-AF 2015–2016, the data collection period spanned one year, from October 2015 to September 2016, to account for seasonal variation. Trained nutritionists conducted two non-consecutive dietary assessment interviews, 8–15 days apart, using a validated software, the eAT24 module of the You eAT&MOVE platform [23]. The dietary assessment method used for adults was two 24-h recall questionnaires. The participants were asked to describe the food items, recipes, and dietary supplements they consumed in detail. This information was recorded using the FoodEx2 classification system [24]. The quantification was performed through a picture book [25] or validated standard units and household measurements. Recipes were disaggregated into single food items, which were then categorised into food groups and subgroups, used to calculate the nutritional intake.

### Adherence to the EAT-Lancet Planetary Health Diet (PHDiet)

To assess adherence to the PHDiet, the PHDiet referential from Willet et al. [5], particularly the energy contribution of each diet component, was used for this analysis. The percentage of the adult population below and above the recommended ranges for each PHDiet component was calculated to estimate the adherence to the PHDiet.

Moreover, a PHDiet score was calculated for each participant using an adapted scoring system based on the Eat Lancet recommendations, previously developed by Cacau et al. [26].The main modifications are:

(1) The cutoffs for scoring the different components were different in some cases: for fruits and vegetables, we considered the minimum range value and the maximum presented in the Eat-Lancet summary report; for pulses and nuts, we used the maximum range values.

(2) Another difference was that we did not use the dark green and red vegetable ratios in this paper, as the Planetary Health Diet from the summary report did not include this information [29].

For calculating the PHDiet score, the individual 2-day average energy contribution (EC, as % of the Total Energy Intake, TEI) of each PHDiet component was considered, and a score between 0 and 10 was attributed. The scoring system for each component is described in Table 1. The final PHDiet score was calculated as the sum of subscores from all components.

**Table 1:** Daily energy intake contributions (EC%) for the Planetary Health Diet (PHDiet) components and respective scoring system

Dietary components	Scoring system	Planetary Health Diet
		Energy contribution (EC%)^1^
		Mean (recommended range)
Whole grains	0 < EC% < 60 → proportional score 0–10	32.4 (0.0–60%)
Tubers	EC% = 0 → score: 0 EC% > 3.1 → score: 0 0 < EC% ≤ 1.6 → proportional score 0–10 1.6 ≤ EC% ≤ 3.1 → proportional score 10–0	1.6 (0.0–3.1)
Vegetables	EC% < 2.1 → score: 0 2.1 < EC% ≤ 6.2 → proportional score 0–10 EC% > 6.2 → score: 10	3.1 (2.1–6.2)
Fruit	EC% < 2.5 → score: 0 2.5 < EC% ≤ 7.6 → proportional score 0–10 EC% > 7.6 → score: 10	5.0 (2.5–7.6)
Dairy	EC% = 0 → score: 0 EC% > 12.2 → score: 0 0 < EC% ≤ 6.1 → proportional score 0–10 6.1 ≤ EC% ≤ 12.2 proportional score 10–0	6.1 (0.0–12.2)
Red Meat	EC% = 0 → score: 10 0 < EC% ≤ 2.4 → proportional score 10–0 EC% > 2.4 → score: 0	1.2 (0.0–2.4)
White Meat	EC% = 0 → score: 10 0 < EC% ≤ 5.0 → proportional score 10–0 EC% > 5.0 → score: 0	2.5 (0.0–5.0)
Eggs	EC% = 0 → score: 0 EC% > 1.5 → score: 0 0 < EC ≤ 0.8→ proportional score 0–10 0.8 ≤ EC% ≤ 1.5 → proportional score 10–0	0.8 (0.0–1.5)
Fish and Seafood	EC% = 0 → score: 0 EC% > 5.7 → score: 0 0 < EC% ≤ 1.6→ proportional score 0–10 1.6 ≤ EC% ≤ 5.7 → proportional score 10–0	1.6 (0.0–5.7)
Pulses	EC% = 0 → score: 0 0 < EC% ≤ 15.1 → proportional score 0–10 EC% > 15.1 → score: 10	11.3 (0.0–15.1)
Nuts	EC% = 0 → score: 0 0 < EC% ≤ 17.5 → proportional score 0–10 EC% > 17.5 → score: 10	11.6 (0.0–17.5)
Added fat—unsaturated oils	EC% < 7.1 → score: 0 EC% > 28.3 → score: 0 7.1 < EC% ≤ 14.1→ proportional score 0–10 14.1 ≤ EC% ≤ 28.3 → proportional score 10–0	14.1 (7.1–28.3)
Added fat—saturated oils	0 < EC% ≤ 3.8 → proportional score 10–0 EC% > 3.8→ score: 0	3.8 (0.0–3.8)
Added sugar	0 < EC% ≤ 4.8 → proportional score 10–0 EC % > 4.8→ score: 0	4.8 (0.0–4.8)

^1^Calculated from the scientific targets for a planetary health diet proposed by Willet et al. [5]

#### Estimating the PHDiet score components using IAN-AF 2015 - 2016 data

The dietary components from the EAT-Lancet reference diet [5] encompass both food group and nutrient components, which are detailed in Table 1. For food group components, each food item reported in IAN-AF 2015–2016 was categorised accordingly (categories: *Whole grains, Tubers, Vegetables, Fruit, Dairy, Red Meat, White Meat, Eggs, Fish and Seafood, Pulses, Nuts*). For nutrient components, a slightly different methodology was used, as explained below.

Some food items reported in the survey, such as beverages and processed foods, did not fit these EAT-Lancet reference diet components. Nevertheless, these foods were considered to calculate the nutrient group components from the EAT-Lancet reference diet [5]: *Added Fat—Unsaturated Oils, Added Fat—Saturated Oils, and Added Suga*r. To estimate the amount of *Added Fats*, we considered the fat content of food items categorised as fats and oils, such as olive oil, vegetable oil, butter, or margarine, which participants reported using in recipes or food items (such as bread). Additionally, we considered the total fat content in processed foods like cakes, cookies, and snacks. A saturated-to-total fat content ratio approach was considered to classify saturated and unsaturated added fats distinctively. Oils, fats, and processed foods with ratios equal to or higher than the ratio of palm oil (saturated/total fat = 0.48) were classified as saturated, while those below this threshold were considered unsaturated. To estimate *Added Suga*r to each food item, an added sugar content was attributed, based on the total sugar content and according to different steps: (a) 0 g of added sugar attributed to foods with 0 g of total sugar and to unprocessed or minimally processed with no added sugars; (b) 100% of added sugar to foods that contained minimal amounts of naturally occurring sugars; (c) added sugar content was calculated based on standard recipes available or on the comparison with the total sugar content of the unsweetened variety; d) 50% of added sugar if an estimation of added sugars was impossible from the previous steps. More details on the complete methodology for estimating added sugars, based on a systematic approach proposed by Louie et al. [27], can be consulted in another publication [28].

### Construct validity of the PHDiet score

Three main hypotheses were tested to assess the validity of the score to measure adherence to the PHDiet, according to the characteristics of this dietary pattern [5]:

1. Higher PHDiet scores reflect higher diet quality, measured through the Healthy Eating Index (HEI);
2. Higher PHDiet scores reflect lower environmental impact, measured with a composite metric of GHGE and LU from the SHARP-ID;
3. Higher PHDiet scores should reflect lower levels of animal protein intake.

#### Diet quality: Healthy Eating Index (HEI)

The Healthy Eating Index (HEI) used in this study consists of nine food groups. The consumption of each food group is measured in grams and scored between 1 and 4 points based on the distribution quartiles by age group. Higher consumption of healthy food groups (“fruit and vegetables”, “cereals and potatoes”, “fish, white meat, and eggs”, and “dairy”) receive higher scores (i.e., max 4 points), while lower consumption receives lower scores (i.e., min 1 point). On the other hand, higher consumption of unhealthy food groups (“red and processed meat”, “salty snacks”, “sweetened beverages”, “sugar and honey”, and “sweets”) receive lower scores (i.e., min 1 point), while lower consumption receives higher scores (i.e., max 4 points). The final HEI score for an individual is the sum of all food group scores, ranging from 9 to 36, with higher scores indicating healthier eating patterns. This HEI was based on the World Health Organization’s (WHO) dietary recommendations [30] and has been previously described in the IAN-AF population [19].

#### Environmental impact: greenhouse gas emissions and land use)

The environmental impact of each participant’s diet was estimated using the SHARP-Indicators Database (SHARP-ID) [31]. For every food item reported in the survey, we attributed a value for each indicator (greenhouse-gas-emissions—GHGE and land-use—LU) using the FoodEx2 classification system as the linkage key. We then estimated the daily individual impacts based on the amount of each food consumed per individual per day. Further details and the complete methodology for assessing the dietary environmental impacts of the Portuguese population are published elsewhere [32].

#### Animal protein

The amount of total protein in the foods reported in IAN-AF 2015–2016 was retrieved from the Portuguese Food Composition Table [33] and then classified as Animal Protein or Vegetable protein as described below:

- For animal-based foods, 100% of the protein was considered animal protein;
- For plant-based foods, 100% of protein was considered vegetable protein (0% animal protein).
- For composite foods with animal- and vegetable-based protein-source ingredients, the animal and vegetable proteins were estimated based on the proportion of the different ingredients in the final product and their respective protein amounts.

### Socioeconomic, lifestyle, health-related factors and macronutrients associated with adherence to PHDiet

The socioeconomic variables from the IAN-AF 2015 2016 participants included in this study were sex, age group, highest completed educational level, food insecurity status (referring to the perceived limited or uncertain availability of nutritionally adequate and safe foods or limited or uncertain ability to acquire acceptable foods in socially acceptable ways [34]), and degree of urbanisation (predominantly urban, moderately urban, and predominantly rural), given by the individuals’ residence parish, according to the Portuguese National Statistics [35]. Health-related variables included a previous diagnosis of chronic disease, and body mass index (BMI), which was categorised based on WHO cut-offs for adults (under/normal weight: < 18.5–24.9 kg/m^2^; overweight: 25.0–29.9 kg/m^2^; and obese: > 30.0 kg/m^2^) [36]. Physical activity level, measured by the International Physical Activity Questionnaire (IPAQ) short-form [37, 38], was used as a lifestyle-related variable.

Moreover, the macronutrient intake adjusted for 2500 kcal was estimated for different levels of adherence to the PHDiet using the Portuguese Food Composition Table [33]information.

### Statistical analysis

All analyses presented in this paper were conducted in R software version 3.6.2[39].

The average estimated PHDiet scores and respective 95%CI for the Portuguese population and by population groups were computed considering the complex sampling design, using the weighting of the sample to ensure national representativeness for the distribution of the Portuguese population, using the library “survey” from R [40].

The PHDiet score was categorised in tertiles, and multivariable-adjusted multinomial regression models were used to test the construct validity and associations between the score and the socioeconomic and health-related variables. The models used to test these assumptions were adjusted for sex and age group.

To check whether different PHDiet levels of adherence presented different macronutrient intake, the nutrients’ energy-adjusted average (per 2500) for the lowest and highest PHDiet terciles were computed, and two-sample t-tests were used to assess significant differences.

Sensitivity analyses were conducted at the PHDiet components subscores to explore the results, whether evaluating the average subscores per category of the determinant variables or applying the multinomial logistic regression models considering the terciles of the PHDiet subscores in the case of the construct validity analysis.

The average usual TEI contribution of each dietary component of the PHDiet was assessed from the two-day assessment using the 1-Part model for daily-consumed foods or food components as a fractional polynomial of age from the SPADE software [41] to statistically correct for within-person variation.

All results with p-values < 0.05 were considered statistically significant.

## Results

### Adherence to the PHDiet

When comparing the recommended intake ranges of the PHDiet components with the intake data from IAN-AF 2015–2016, Among the dietary components from the PHDiet, red meat stands out with more than 98% of the population having a daily consumption above the recommended, as shown in Table 2. White meat and added sugars are also consumed above the recommended ranges in a high proportion of the population (> 60%). On the contrary, the consumption of pulses, nuts and whole grains is below the average level of the range recommended in the PHDiet for more than 99% of the population. Vegetables present a consumption below the minimum recommended for 41% of the population.

**Table 2:** Daily energy intake contributions (%TEI) for the Planetary Health Diet components observed in the Portuguese population (n = 3852), and the proportion of the population below or above the recommended cut-offs

Dietary components	IAN-AF 2015–2016			
	Energy contribution^1^	Proportion of the population		
	Mean	< Min recommended	< Average^2^	> Max recommended
	***%***			
Whole grains	2.4		99.9	< 0.001
Tubers	4.4	–	6.5	67.6
Vegetables	2.6	40.6	–	1.2
Fruit	6.9	13.3	–	38.0
Dairy	8.7	–	39.2	22.5
Red meat	10.1	–	0.2	98.0
White meat	6.7	–	3.4	67.4
Eggs	1.4	–	33.6	34.3
Fish and seafood	4.7	–	6.6	29.4
Pulses	2.8	–	99.2	0.2
Nuts	1.0	–	99.6	0.1
Added fat—unsaturated oils	11.2	10.4	–	0.01
Added fat—saturated oils	2.9	–	73.6	26.4
Added sugar	6.8	–	37.0	63.0

^1^Usual energy contribution, calculated with correction for within-person variability

^2^Within the range, considered when the minimum cut-off from the Planetary Health Diet proposed by Willet et al. [5] is 0.0%

Table 3 shows the average score and 95% confidence intervals for different population groups. Theoretically, the PHDiet score could vary between 0 and 140 points. The overall average score in the Portuguese Population was 36.0 (95% CI: 35.4–36.6), ranging from 9.7 (percentile 1%) to 67.7 (percentile 99%), as shown in Table 3. The average PHDiet score among females is 37.2 (95%CI: 36.4; 38.0), while among males, it is 34.7 (95%CI: 33.8; 35.6). The average PHDiet among adults (18–64 years) is 34.5 (95%CI: 33.7; 35.2), while among older individuals (≥ 65 years) is 41.8 (95%CI: 40.5; 43.1).

**Table 3:** Average Planetary Health Diet Score (PHDiet-score) in the Portuguese population, the Portuguese National Dietary Survey (IAN-AF 2015–2016), n = 3852

	n (%)	Average PHDiet-score
		Mean (95% CI)
Overall	3852 (100)	36.0 (35.4; 36.6)
PHDiet		
Low (T1)	1284 (33.3)	21.9 (21.5; 22.4)
Intermediate (T2)	1284 (33.3)	35.0 (34.7; 35.2)
High (T3)	1284 (33.3)	50.2 (49.5; 50.9)
Sex		
Female	2032 (52.8)	37.2 (36.4; 38.0)
Male	1820 (47.2)	34.7 (33.8; 35.6)
Age group		
Adults (18–64 years)	3102 (80.5)	34.5 (33.7; 35.2)
Elderly (≥ 65 years)	750 (19.5)	41.8 (40.5; 43.1)
Educational level		
≤ 6 years	1342 (34.9)	38.6 (37.6; 39.6)
6–12 years	1629 (42.3)	33.7 (32.8; 34.6)
> 12 years	876 (22.8)	36.7 (35.6; 37.9)
Degree of urbanisation		
Predominantly urban	2803 (72.8)	36.3 (35.7; 36.9)
Moderately urban	680 (17.6)	34.8 (33.1; 36.4)
Predominantly rural	369 (9.6)	35.1 (33.2; 36.9)
Food insecurity		
No	3448 (89.7)	36.3 (35.6; 36.9)
Yes	397 (10.3)	33.6 (31.9; 35.3)
BMI class		
Normal	1340 (36.6)	34.1 (33.0; 35.3)
Overweight	1373 (37.5)	37.1 (36.0; 38.2)
Obese	950 (25.9)	36.6 (35.3; 38.0)
Chronic disease		
No	2201 (57.1)	33.9 (33.0; 34.8)
Yes	1651 (42.9)	38.7 (37.7; 39.6)
IPAQ level		
Inactive	1625 (43.4)	35.2 (34.3; 36.1)
Minimally active	1176 (31.5)	36.5 (35.4; 37.6)
Very active	937 (25.1)	36.4 (34.9; 37.8)

### Construct validity of PHDiet score

Table 4 presents the results of the analysis examining the construct validity through the associations (OR and respective 95%CI) between PHDiet-score and health (HEI), environmental (SHARP-ID indicators GHGE and Land Use) and dietary (animal protein) parameters. All hypotheses were confirmed for the overall score. Namely, negative associations were found for HEI (Low vs High: OR = 0.70, 95% CI 0.68–0.72; Intermediate vs High: OR = 0.83, 95% CI 0.80–0.85), indicating that the highest levels of adherence to the PHDiet were associated with a healthier diet. In contrast, the environmental impact indicators estimated from SHARP-ID were significantly higher for the low and intermediate PHDiet-score categories (Low vs High GHGE: OR = 1.31, 95% CI 1.26–1.37; Intermediate vs High GHGE: OR = 1.1.17, 95% CI 1.12–1.22; Low vs High LU: OR = 1.25, 95% CI 1.21–1.29; Intermediate vs High LU: OR = 1.15, 95% CI 1.11–1.18), showing that individuals with higher adherence to the PHDiet have diets with lower dietary GHGE and LU impacts. Furthermore, increased animal protein intake was associated with higher odds of having lower and intermediate PHDiet-scores (per each 10 g/day increase: Low vs High: OR = 1.11, 95% CI 1.06–1.16; Intermediate vs High: OR = 1.04, 95% CI 1.00–1.09).

**Table 4:** Construct validity of Planetary Health Diet (PHDiet) score, based on PHDiet terciles (T1-T3) estimated for the Portuguese National Dietary Survey (IAN-AF 2015–2016), n = 3852

Parameters	Hypothesis^1^	Low (T1) vs high (T2) PHDiet-score		Intermediate (T2) vs high (T3) PHDiet-score	
		OR^2^	95% CI	OR^2^	95% CI
Healthy eating index (HEI)	Increasing HEI leads to lower odds of having lower PHDiet adherence (T1 vs T3),	**0.70**	**0.68; 0.72**	**0.83**	**0.80; 0.85**
Environmental impact—land use (m^2^/year)	Increasing dietary environmental impact (LU and GHGE) leads to higher odds of having lower PHDiet adherence (T1 vs T3),	**1.25**	**1.21; 1.29**	**1.15**	**1.11; 1.18**
Environmental impact—GHGE (kgCO_2_eq/day)		**1.31**	**1.26; 1.37**	**1.17**	**1.12; 1.22**
Animal protein (per each 10 g/day)	Increasing animal protein intake leads to higher odds of having lower PHDiet adherence (T1 vs T3),	**1.11**	**1.06; 1.16**	**1.04**	**1.00; 1.09**

^1^According to the rationale of Willet et al. [5]

^2^Adjusted for sex and age group. Reference category: High PHDiet score

Statistical significant results (*p*-value<0.05) are presented in bold

A more detailed sensitivity analysis was conducted at the PHDiet components subscores against the variables used to evaluate construct validity, as shown in Figure S1.1 from Supplementary Material 1. In general, most results were consistent with the overall PHDiet score. The most pronounced differences were found for the HEI, where some subscores presented a result contrary to the overall PHDiet score. Namely, it was found that higher subscores for White meat, Eggs and Tubers were associated with lower values of the HEI.

### Associations between PHDiet-score and sociodemographic factors

**Fig. 1:**
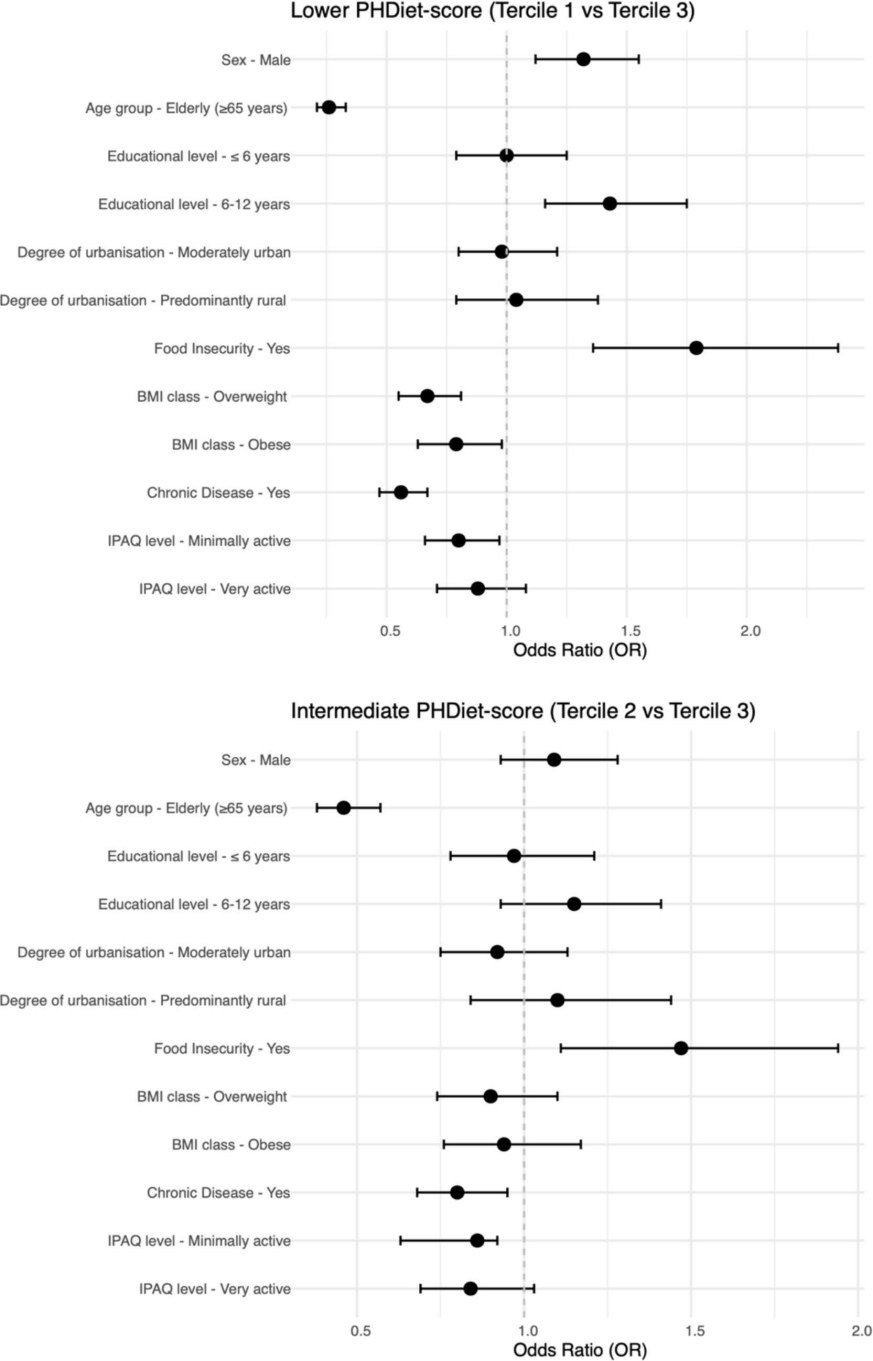
Odds ratio for the associations of sociodemographic and health-related factors with PHDiet-score tertiles in the Portuguese National Dietary Survey (IAN-AF 2015–2016), n = 3852.*. *Multinomial logistic regression models adjusted for sex, age and educational level. The error bars indicate the 95%CI. Reference PHDiet tercile: “High PHDiet score. Variables reference categories—sex: female, age group: adults, educational level: > 12 years, degree of urbanisation: predominantly urban; food insecurity: no; bmi class: normal weight; chronic disease: no; IPAQ level: inactive

Figure 1 and Table S1.1. present the results of the multivariable-adjusted multinomial regression models. After adjusting for potential confounders (sex, age group and educational level) and using the highest category of PHDiet score as reference, men (OR = 1.32, 95% CI 1.12–1.55), individuals with intermediate education level (6–12 years: OR = 1.43, 95% CI 1.16–1.75, ref: higher education) and suffering from food insecurity (OR = 1.79, 95% CI 1.36–2.38) presented higher odds of having lower PHDiet score. The opposite was found for older people (OR = 0.26, 95% CI 0.21–0.33), overweight (OR = 0.67, 95% CI 0.55–0.81) or obese individuals (OR = 0.79, 95% CI 0.63–0.98), people diagnosed with a chronic disease (OR = 0.56, 95% CI = 0.47–0.67) and with intermediate physical activity levels (OR = 0.80, 95% CI 0.66–0.97; ref: inactive).

Further insights on these associations can be consulted in Supplementary Material 1, Figure S2 to S16, where a graphical representation of the average PHDiet subscores per category of the associated factors is presented for each PHDiet component.

**Fig. 2:**
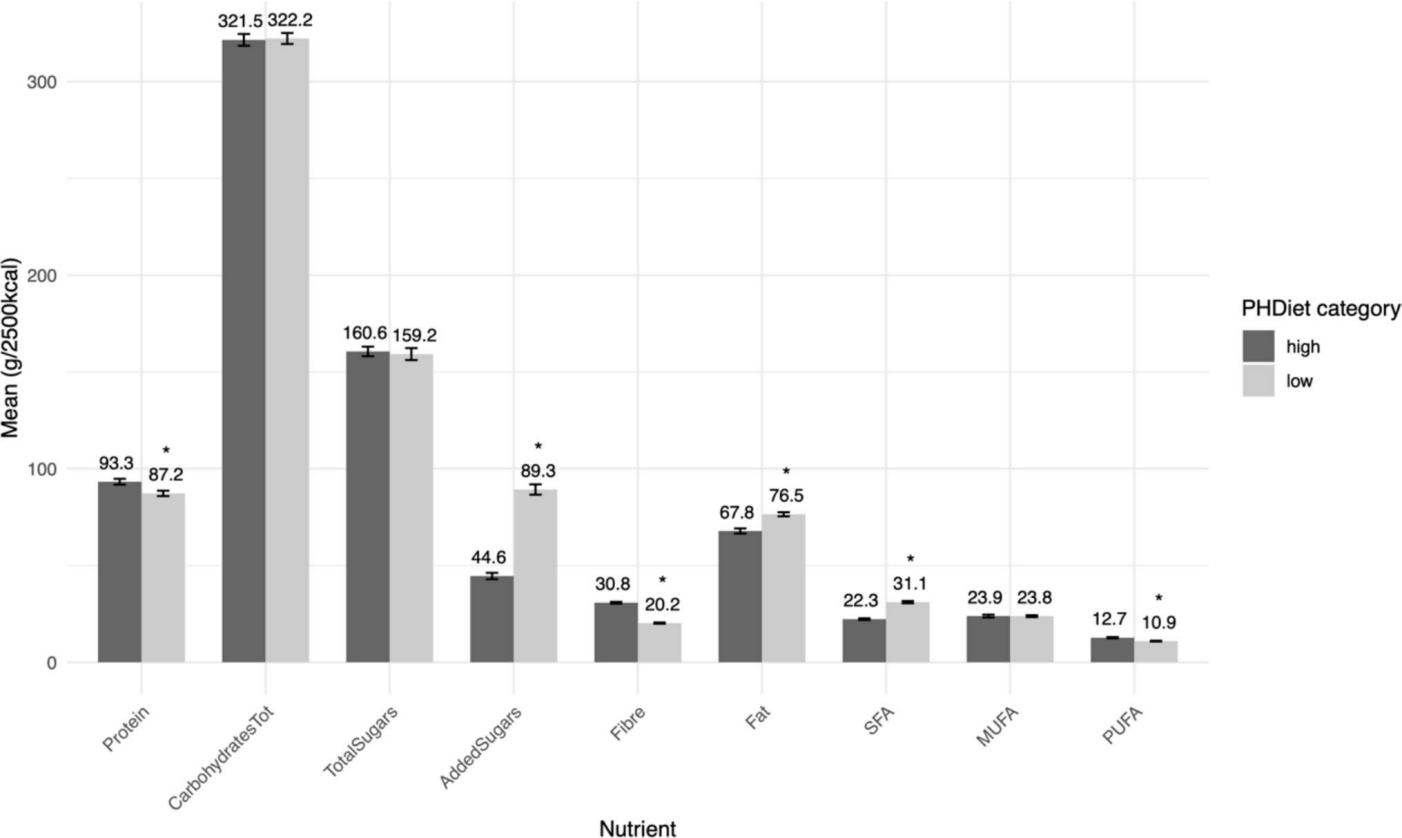
Average nutrient intakes (in g/2500 kcal) by PHDiet tercile (Low: T1; High: T3) in the Portuguese National Dietary Survey (IAN-AF 2015–2016), n = 3852. The average PHDiet score was 21.9 for T1 and 50.2 for T3. *Significant differences between high and low terciles (two-sample t-test, p-value < 0.05)

### PHDiet-score and nutrient intake

When comparing the average nutrient intakes of different levels of adherence to the PHDiet, it was possible to find that compared to those from the first tercile (lowest adherence), individuals from the third tercile had significantly higher intake (more than 10% difference and p-value < 0.05) of fibre (30.8 g/2500 kcal vs 20.2 g/2500 kcal) and polyunsaturated fatty acids (PUFA) (12.7 g/2500 kcal vs. 10.9 g/2500 kcal); and lower intake of added sugars (44.6 g/2500 kcal vs 89.3 g/2500 kcal), total fat (67.8 g/2500 kcal vs 76.5 g/2500 kcal) and saturated fat (22.3 g/2500 kcal vs 31.1 g/2500 kcal) (see Fig. 2).

## Discussion

This study evaluated the adherence of the Portuguese population to a PHDiet score, which was based on the guidelines from the EAT-Lancet Commission on Food, Planet, Health. Several hypotheses were tested to assess the construct validity of the global score and subscores. In most cases, the hypotheses were confirmed, showing this score as a valid measure of a healthy and sustainable dietary pattern, as the Planetary Healthy Diet proposed by Willet et al. [5]. First, we hypothesised that the PHDiet should be consistent with other measures of diet quality. Accordingly, the results showed that increasing scores for a HEI based on the WHO recommendations for a healthy diet [30] were associated with higher PHDiet scores. Nevertheless, some inconsistencies were found when considering the PHDiet subscores. Namely, higher subscores for White meat, Eggs and Tubers were associated with lower values of the HEI. This was somehow expected since these food groups were rated as “positive” or “healthy” components on the HEI (i.e., higher intakes consistently higher index). Still, in the PHDiet, the value intakes score gradually higher up to the recommended range average but then, above that point, score gradually lower. This underscores the challenge to optimise the health and sustainable aspects of diets, even when considering a reference dietary pattern such as the EAT-Lancet Planetary Health Diet.

Regarding the environmental domain, it was found that increasing dietary environmental impacts were associated with higher odds of having low PHDiet scores, i.e., low adherence to the recommendations. In other words, individuals with higher adherence to the PHDiet presented lower dietary GHGE and Land Use. This was also expected, as in general, foods that scored positively in the PHDiet, such as vegetables, fruits, and overall plant-based foods, present lower environmental impacts than animal-based ones, according to different data sources [31, 42, 43]. Moreover, other studies have found a high potential for reducing dietary greenhouse gas emissions and land use with the adoption of the PHDiet in Western populations [14, 44].

Given that the PHDiet score overall scores plant-based foods higher than animal-based ones, animal protein intake was the last hypothesis tested for the score’s construct validity. Again, the results were in line with what was expected, and higher intakes of animal protein were associated with higher odds of having a low PHDiet score. As previously found in other studies evaluating PHDiet adherence [26, 45]. Besides animal protein, other nutrients were assessed regarding the PHDiet. The findings point to significantly higher intakes of fibre and PUFA and lower intakes of added sugars, total fat and saturated fatty acids for increased levels of adherence to the PHDiet, suggesting better profiles for several macronutrients (such as fibre and added sugars) for PHDiet higher adherence levels, similarly to other studies [12, 26, 46, 47].

When assessing the average PHDiet score in the Portuguese population, the findings of this study show an overall low score result (i.e., around 26% of the maximum score), with several dietary components characterising this dietary pattern presenting a high proportion of individuals with intakes outside the recommended ranges. Specifically, dietary patterns in Portugal tend to exceed the PHDiet recommended consumption levels for meat (red and white) and added sugars while falling short of the recommended intake for pulses, nuts, whole grains, and vegetables. These results support the relevance and potential impact of a concerted public health policy to promote the adoption of more sustainable and healthier dietary patterns at the population level, particularly targeting these specific foods.

Previous studies evaluating other populations’ adherence to the Planetary Health Diet have also found lower adherence levels [10, 12, 47]. Stubbendorff et al.[12] found low adherence to the Planetary Health Diet in a Swedish cohort and shallow points of adherence to foods like “Beef”, “Pork”, “Grains”, Nuts and Pulses, which were also highlighted in this study as PHDiet components with a high prevalence of individuals outside the recommended ranges. Moreover, when looking specifically into the dietary patterns of the Portuguese population, other studies focusing on adherence to healthy and sustainable dietary patterns, such as the Mediterranean diet, also showed relatively low adherence in Portugal [16, 48, 49], supporting these findings. A recent study has shown that participants with lower adherence to the Mediterranean dietary pattern have lower expenditures on nuts, pulses, and vegetables while having higher expenditures on meat than those in the higher adherence group [50].

In this study, sociodemographic factors such as gender, education level, and food insecurity were shown to play significant roles in PHDiet adherence, with men, intermediate-educated individuals, and those facing food insecurity being more likely to have lower scores. Previous research evaluating adherence to the EAT-Lancet recommendations and other healthy dietary patterns and their association with several socioeconomic factors has shown consistent results at the national [49–51] and international levels [9, 11, 12, 45, 47, 52]. Namely, females generally adhere more to healthier dietary patterns than men [51, 53–56], which may be explained by several reasons, such as higher health consciousness and a heightened interest in nutritional topics [57, 58]. The PHDiet subscores for Whole grains, Vegetables, Fruits, and Red Meat were significantly higher among females, while Dairy and Added sugars were higher among males (Figure S1.2 to S1.15). Socioeconomic status is also expected to impact adherence and access to healthier diets. Food insecurity status has been associated with low adherence to the Mediterranean diet in a national cohort study, in which the sample is considered representative of the Portuguese population [59]. In the same study, individuals from food-insecure households presented reduced consumption of vegetables, fruit, fish or seafood, and nuts. These findings suggest a parallelism between the Mediterranean dietary pattern and the PHDiet measured in the current study. This study found that people suffering from food insecurity had significantly lower average PHDiet subscores for Tubers, Vegetables and Fruits. The other subscores did not significantly differ depending on the food insecurity status (Figure S1.2 to S1.15).

In this study, overweight and obese individuals and those with chronic diseases presented higher adherence to the PHDiet, even after adjusting for possible confounders (sex, age, and educational level). These unexpected findings may arise due to the study’s cross-sectional design and the subsequent possibility of reverse causality due to dieting or specific restrictive dietary patterns. It can also be a result of misreporting, as previous research has shown that obese individuals are more prone to under-reporting [60].

The cross-sectional design of this study is, then, a relevant constraint that limited the possibility of studying associations of the PHDiet score with health outcomes due to the possibility of reverse causality and limited health outcome variables available. Moreover, the dietary intake was assessed with self-reporting methods, which could lead to bias and misreporting. Nevertheless, the IAN-AF 2015–2016 methodology followed standardised protocols according to the European guidelines from the EU Menu project [22] and included a large and representative sample of Portuguese adults, strengthening the methodology. Additionally, the use of multiple 24 h-recalls applied by trained interviewers with a background in dietetics and nutrition permitted the collection of detailed and accurate food consumption data and the estimation of the usual food intake using SPADE software. This approach narrows the intake distribution by correcting for within-person variability, granting a more precise and less biased estimate of the prevalence of individuals with consumption above or below the recommendations.

This study represents the first attempt to evaluate the Portuguese adult population’s adherence to the EAT-Lancet reference diet. A score based on the EAT-Lancet Commission on Food, Planet, Health recommendations was developed, and the results from the associations with diet quality and environmental parameters supported its construct validity. Nevertheless, some aspects of the scoring system might not be ideal for some food groups in some contexts. For instance, for whole grains, fruits, vegetables, pulses and nuts, the scoring system proposed a gradual score from 0 to 10 for values within the recommended range. Also, 10 points would be attributed to intakes equal to or above the maximum range value. This approach fits the context of this population since there is not a significant proportion of individuals with such high consumption values. However, one might argue that attributing the maximum score would not be the most adequate approach for consumption values above the maximum range limit. Thus, the external validity of this PHDiet could not be confirmed for populations with different dietary patterns. Another limitation of the current version of the proposed PHDiet score is related to the difficulty of achieving the maximum score (i.e., 10 points) for the dietary components that establish a single optimal value, hindering the possibility of attaining full adherence to this score. Nevertheless, in a sensitivity analysis, a second version of the PHDiet score was computed. Instead of considering only one single optimal value for these components, 10 points were attributed to intakes within the range between 0 and the average energy contribution. Overall, this second score version resulted in higher absolute scores. Still, the regression models used to test the construct validity and associations with socioeconomic variables, as well as the nutrient analysis, yielded similar overall results to the main PHDiet score (Supplementary Material 2).

Moreover, despite IAN-AF 2015–2016 being the most recent nationally representative data source, it must be acknowledged that the data collection period was before the launch of the PHDiet recommendations. Thus, a re-evaluation should be done timely once new nationally representative data is available.

## Conclusion and future perspectives

This study showed that the PHDiet score is valid and associated in the expected direction with other measures of diet quality (higher diet quality → higher score) and environmental impact (higher environmental impact → lower score), proven to be a good instrument to be used in further studies for assessing adherence to the PHDiet.

Moreover, the study provided insights into the Portuguese population’s adherence to the PHDiet, revealing overall low levels of adherence to this healthy and sustainable dietary pattern. The dietary components with a higher prevalence of consumption outside the recommended range were meat (high intake), nuts, pulses and whole grains (low intake), supporting the need for a shift in dietary patterns in the Portuguese population focused on substituting, particularly meat for vegetable alternatives (e.g., nuts, pulses), which should be sustained by targeted public health policies, particularly addressing individuals prone to food insecurity to avoid additional inequities. Further longitudinal studies could be used to study the associations between adherence to the PHDiet and health outcomes, using this PHDiet score to sustain the health effects of this dietary pattern.

## Supplementary Information

Below is the link to the electronic supplementary material.

Supplementary file1 (DOCX 2616 KB)

Supplementary file2 (DOCX 454 KB)

## Data Availability

Data from the IAN-AF 2015–2016 study is publicly available and can be requested at https://www.ian-af.up.pt/en/. The datasets used and analyzed during the current study are available from the corresponding author upon reasonable request.
