# Supplementary material for: Adherence to the EAT-Lancet Planetary Health Diet in Portugal and its associations with socioeconomic and lifestyle factors
Source: Eur J Nutr. 2025 Apr 9;64(4):152. doi: 10.1007/s00394-025-03661-6 (PMC11982082; doi:10.1007/s00394-025-03661-6)
Supplement: Supplementary file 1 — Supplementary file1 (DOCX 2616 KB) [file 394_2025_3661_MOESM1_ESM.docx]

**Supplementary material 1**

**Adherence to the Eat-Lancet-Planetary Health Diet in Portugal and its associations with socioeconomic and lifestyle factors**

Catarina Carvalho^1,2,3^, Daniela Correia^1,2,4^, Carla Lopes^1,2,4^, Duarte Torres^1,2,3^

^1^ EPIUnit – Instituto de Saúde Pública, Universidade do Porto, Porto, Portugal.

^2^ Laboratório para a Investigação Integrativa e Translacional em Saúde Populacional (ITR), Porto, Portugal

^3^ Faculdade de Ciências da Nutrição e Alimentação, Universidade do Porto, Porto, Portugal.

^4^ Departamento de Ciências da Saúde Pública e Forenses e Educação Médica, Faculdade de Medicina, Universidade do Porto, Porto, Portugal.

**Table of Contents – Supplementary tables**

[**Table S1.** *Odds ratio for the* multivariable-adjusted multinomial logistic regression analysis for the associations of *sociodemographic* and health-related factors with PHDiet-score tertiles (“low”, “intermediate”, “high”) in the Portuguese National dietary survey (IAN-AF 2015-2016) 3](#_Toc181392361)

**Table of Contents – Supplementary figures**

[**Figure S1.1.** Construct validity models applied to the different PHDiet component subscores. Reference PHDiet tercile: “High PHDiet score” **–** The bars represent the odds of having a low PHDiet component subscores. HEI = Healthy Eating Index; AnimalProtein = Per 10 g animal protein; GHGE = per kg Greenhouse gas emissions; Land use = per m2/y Land use 4](#_Toc189654982)

[**Figure S1.2.** Average PHDiet Component Subscore – **Whole Grains** - per category of the sociodemographic and health-related variables under study. Higher scores represent higher adherence. 5](#_Toc189654983)

[**Figure S1.3.** Average PHDiet Component Subscore – **Tubers** - per category of the sociodemographic and health-related variables under study. Higher scores represent higher adherence. 6](#_Toc189654984)

[**Figure S14.** Average PHDiet Component Subscore – **Vegetables** - per category of the sociodemographic and health-related variables under study. Higher scores represent higher adherence. 7](#_Toc189654985)

[**Figure S1.5.** Average PHDiet Component Subscore – **Fruits** - per category of the sociodemographic and health-related variables under study. Higher scores represent higher adherence. 8](#_Toc189654986)

[**Figure S1.6.** Average PHDiet Component Subscore – **Dairy** - per category of the sociodemographic and health-related variables under study. Higher scores represent higher adherence. 9](#_Toc189654987)

[**Figure S1.7.** Average PHDiet Component Subscore – **Red Meat** - per category of the sociodemographic and health-related variables under study. Higher scores represent higher adherence. 10](#_Toc189654988)

[**Figure S1.8.** Average PHDiet Component Subscore – **White Meat** - per category of the sociodemographic and health-related variables under study. Higher scores represent higher adherence. 11](#_Toc189654989)

[**Figure S1.9.** Average PHDiet Component Subscore – **Eggs** - per category of the sociodemographic and health-related variables under study. Higher scores represent higher adherence. 12](#_Toc189654990)

[**Figure S1.10.** Average PHDiet Component Subscore – **Fish and Seafood** - per category of the sociodemographic and health-related variables under study. Higher scores represent higher adherence. 13](#_Toc189654991)

[**Figure S1.11.** Average PHDiet Component Subscore – **Pulses** - per category of the sociodemographic and health-related variables under study. Higher scores represent higher adherence. 14](#_Toc189654992)

[**Figure S1.12.** Average PHDiet Component Subscore – **Nuts** - per category of the sociodemographic and health-related variables under study. Higher scores represent higher adherence. 15](#_Toc189654993)

[**Figure S1.13.** Average PHDiet Component Subscore – **Added fats – Unsaturated Oils** - per category of the sociodemographic and health-related variables under study. Higher scores represent higher adherence. 16](#_Toc189654994)

[**Figure S1.14.** Average PHDiet Component Subscore – **Added Fat – Saturated oils** - per category of the sociodemographic and health-related variables under study. Higher scores represent higher adherence. 17](#_Toc189654995)

[**Figure S1.15.** Average PHDiet Component Subscore – **Added Sugars** - per category of the sociodemographic and health-related variables under study. Higher scores represent higher adherence. 18](#_Toc189654996)

**Table S1.1.** *Odds ratio for the* multivariable-adjusted multinomial logistic regression analysis for the associations of *sociodemographic* and health-related factors with PHDiet-score tertiles (“low”, “intermediate”, “high”) in the Portuguese National Dietary Survey (IAN-AF 2015-2016), n=3852.

|  | **Multinomial logistic regression model^1^** | |
| --- | --- | --- |
|  | ***Low*** *vs High PHDiet-score* | ***Intermediate*** *vs High PHDiet-score* |
|  | OR **(**95%CI) | OR (95%CI) |
| Overall |  |  |
| Sex |  |  |
| Female | Ref. | Ref. |
| Male | **1.32 (1.12; 1.55)** | 1.09 (0.93; 1.28) |
| Age group |  |  |
| Adults (18-64 years) | Ref. | Ref. |
| Elderly (≥65 years) | **0.26 (0.21; 0.33)** | **0.46 (0.38; 0.57)** |
| Educational level |  |  |
| ≤ 6 years | 1.00 (0.79; 1.25) | 0.97 (0.78; 1.21) |
| 6-12 years | **1.43 (1.16; 1.75)** | 1.15 (0.93; 1.41) |
| >12 years | Ref. | Ref. |
| Degree of urbanisation |  |  |
| Predominantly urban | Ref. | Ref. |
| Moderately urban | 0.98 (0.80; 1.21) | 0.92 (0.75; 1.13) |
| Predominantly rural | 1.04 (0.79; 1.38) | 1.10 (0.84; 1.44) |
| Food Insecurity |  |  |
| No | Ref. | Ref. |
| Yes | **1.79 (1.36; 2.38)** | 1.47 (1.11; 1.94) |
| BMI class |  |  |
| Normal | Ref. | Ref. |
| Overweight | **0.67 (0.55; 0.81)** | 0.90 (0.74; 1.10) |
| Obese | **0.79 (0.63; 0.98)** | 0.94 (0.76; 1.17) |
| Chronic Disease |  |  |
| No | Ref. | Ref. |
| Yes | **0.56 (0.47; 0.67)** | **0.80 (0.68; 0.95)** |
| IPAQ level |  |  |
| Inactive | Ref. | Ref. |
| Minimally active | **0.80 (0.66; 0.97)** | **0.86 (0.63; 0.92)** |
| Very active | 0.88 (0.71 1.08) | 0.84 (0.69; 1.03) |
| 1. Adjusted for sex, age and educational level; Reference category “High PHDiet score” 2. In bold, are highlighted the statistically significant results, based on the 95%CI. | | |


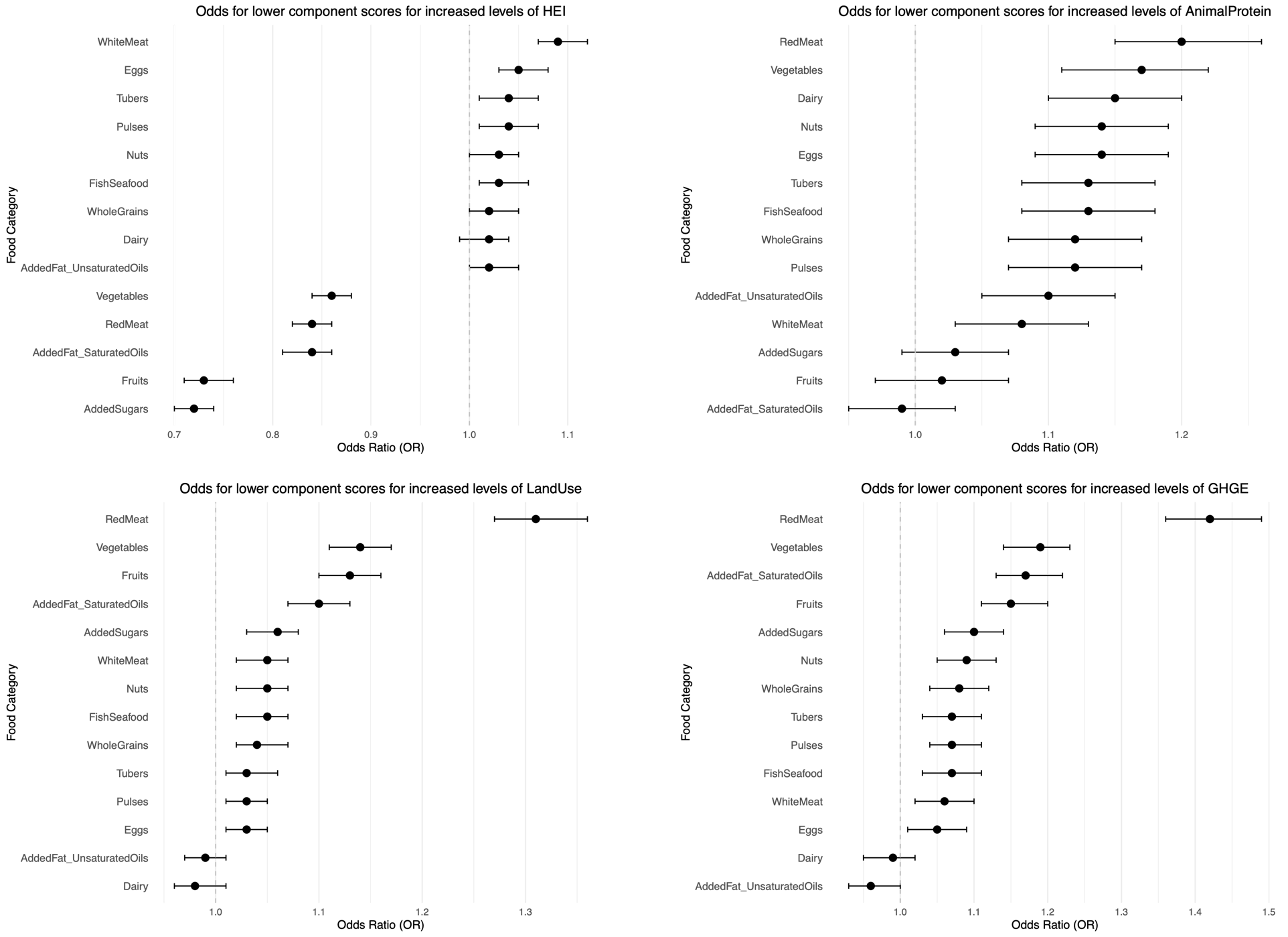


**Figure S1.1.** Construct validity models applied to the different PHDiet component subscores. Reference PHDiet tercile: “High PHDiet score” **–** The bars represent the odds of having a low PHDiet component subscores. HEI = Healthy Eating Index; AnimalProtein = Per 10 g animal protein; GHGE = per kg Greenhouse gas emissions; Land use = per m2/y Land use


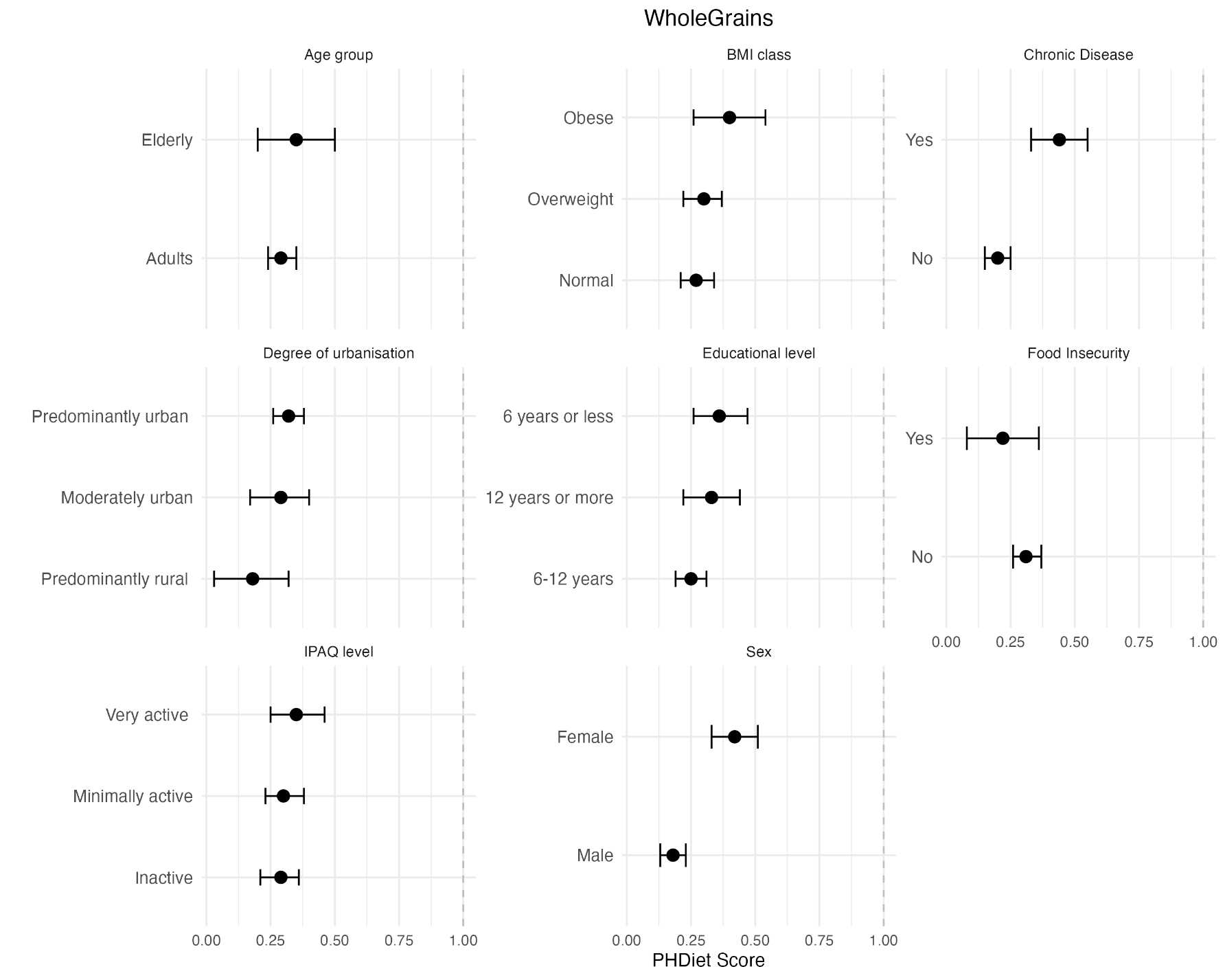


**Figure S1.2.** Average PHDiet Component Subscore – **Whole Grains** - per category of the sociodemographic and health-related variables under study. Higher scores represent higher adherence.


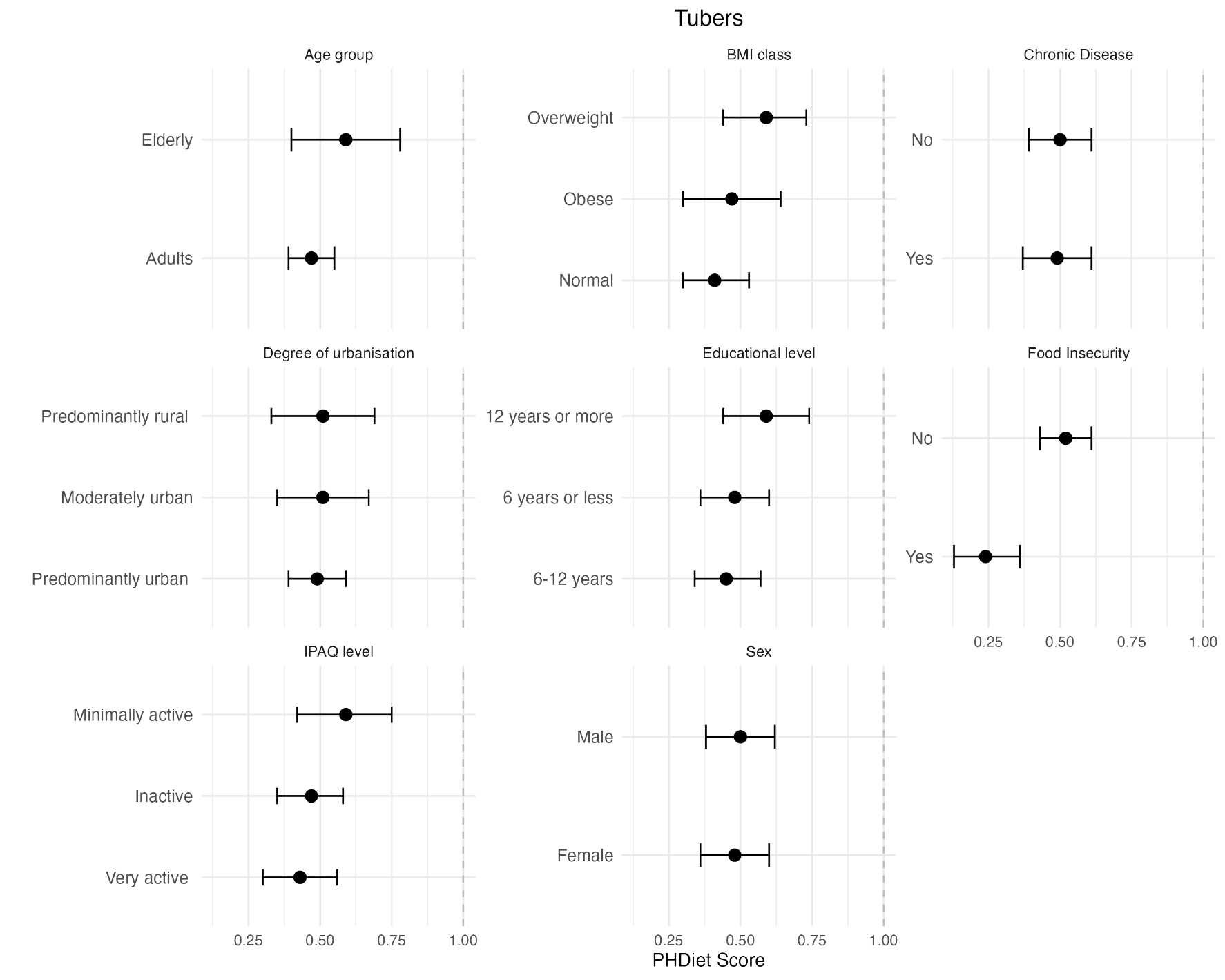


**Figure S1.3.** Average PHDiet Component Subscore – **Tubers** - per category of the sociodemographic and health-related variables under study. Higher scores represent higher adherence.


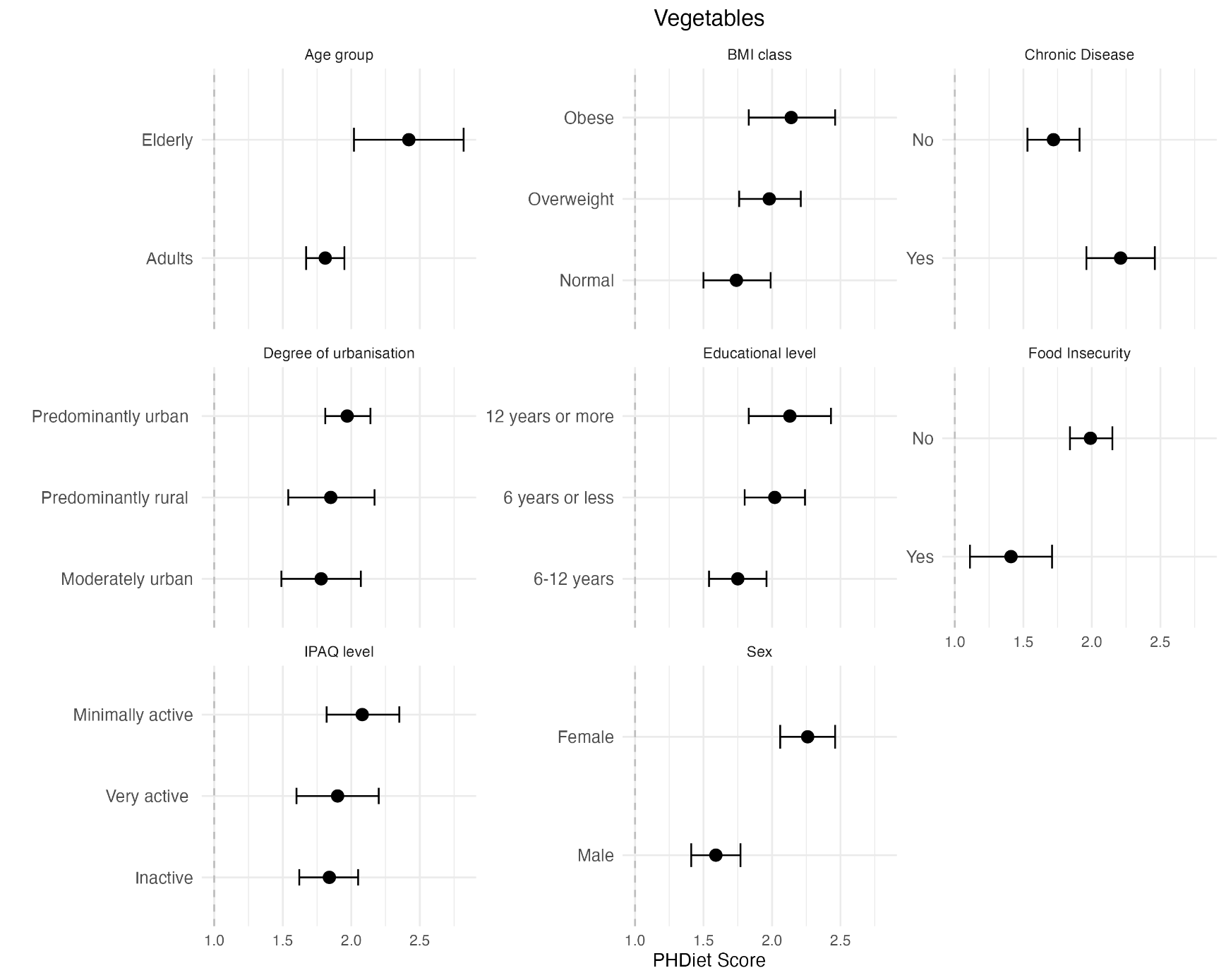


**Figure S14.** Average PHDiet Component Subscore – **Vegetables** - per category of the sociodemographic and health-related variables under study. Higher scores represent higher adherence.


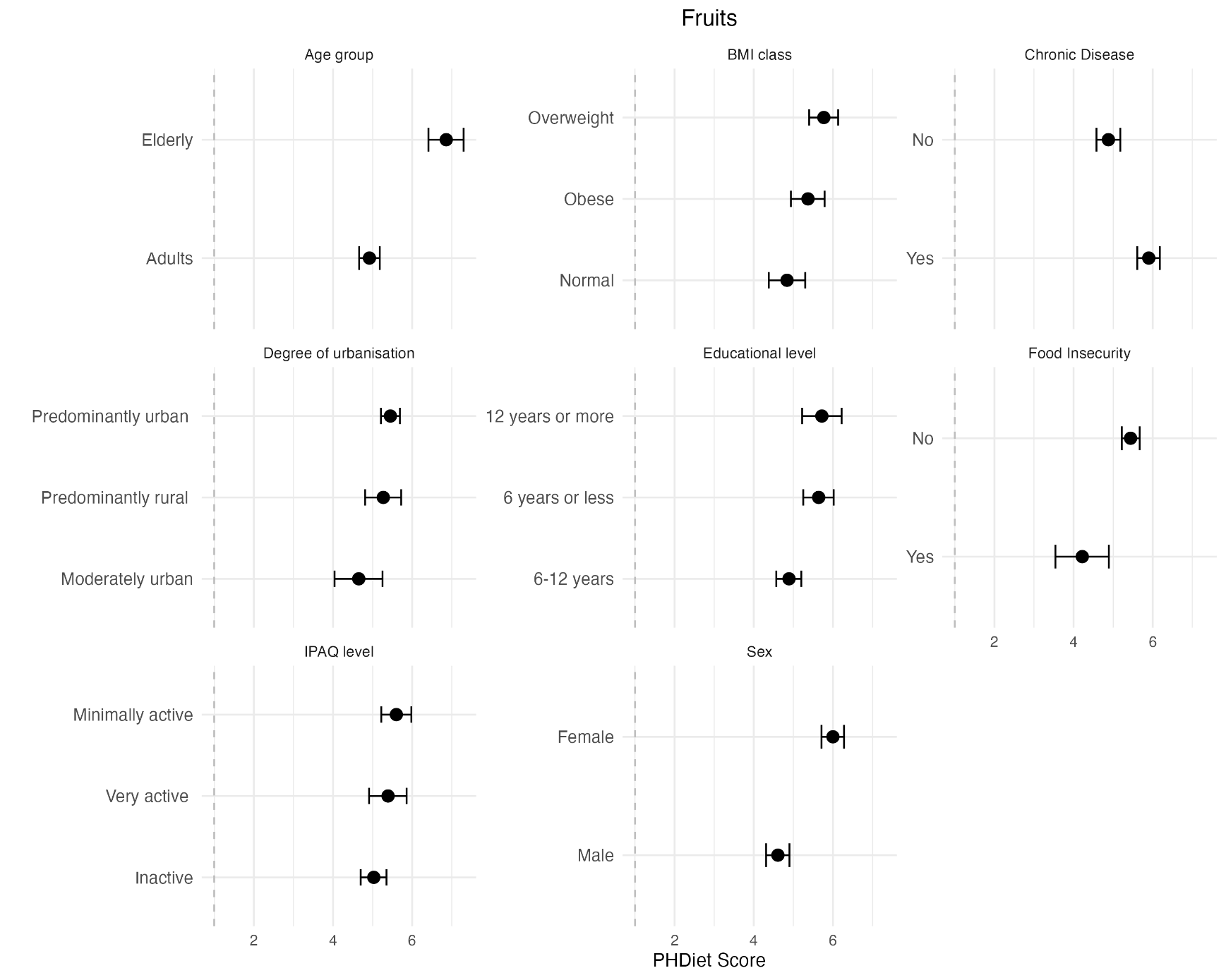


**Figure S1.5.** Average PHDiet Component Subscore – **Fruits** - per category of the sociodemographic and health-related variables under study. Higher scores represent higher adherence.


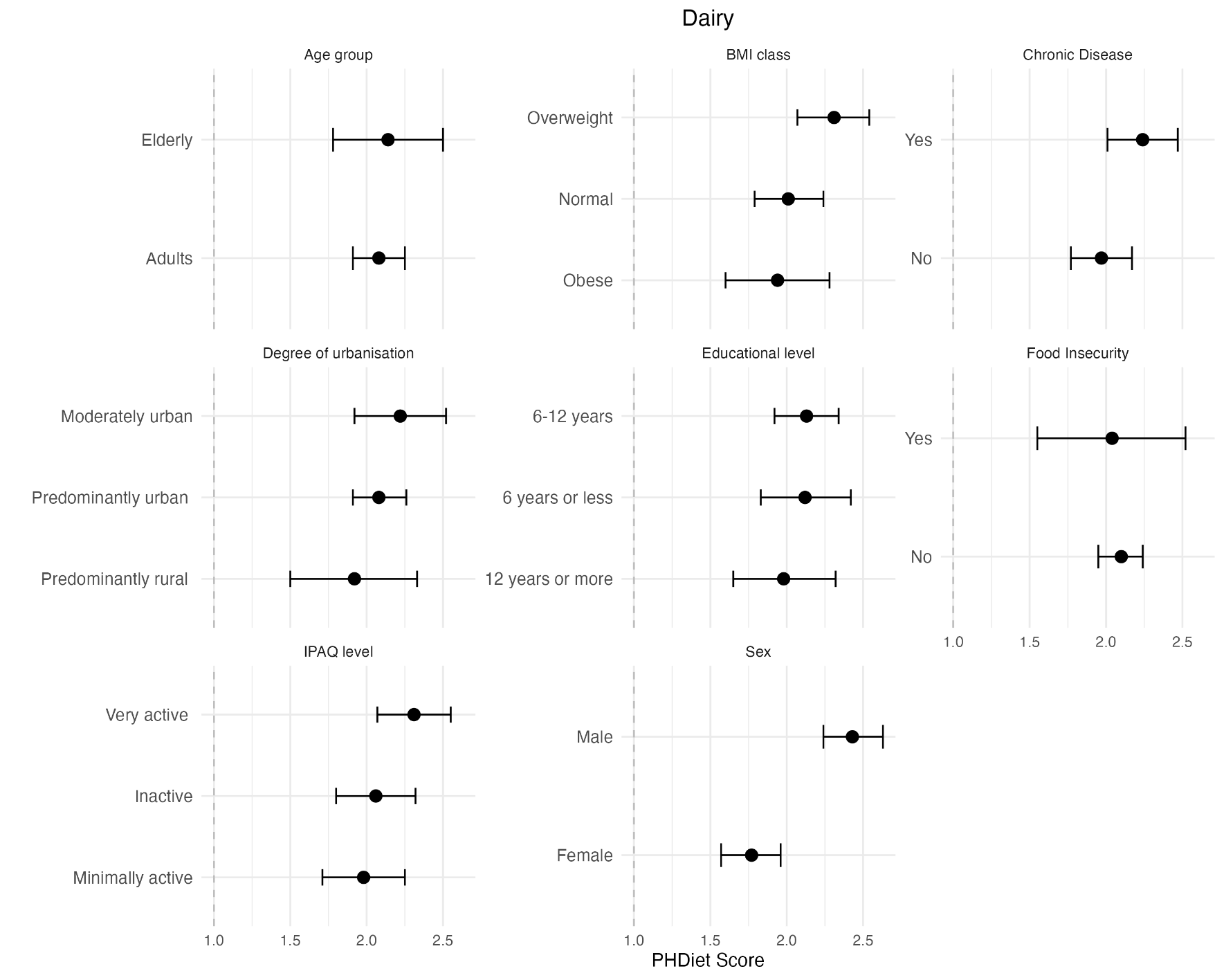


**Figure S1.6.** Average PHDiet Component Subscore – **Dairy** - per category of the sociodemographic and health-related variables under study. Higher scores represent higher adherence.


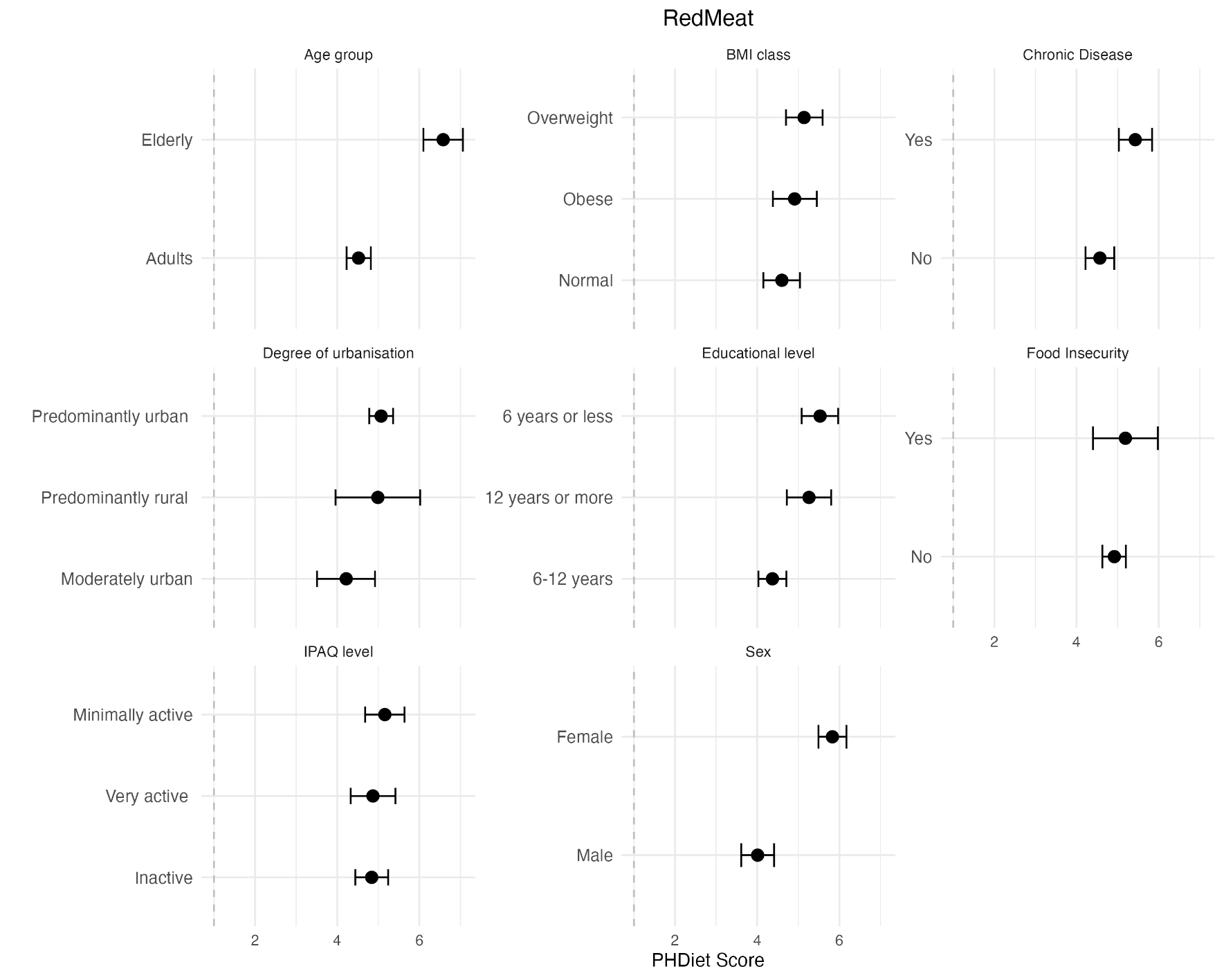


**Figure S1.7.** Average PHDiet Component Subscore – **Red Meat** - per category of the sociodemographic and health-related variables under study. Higher scores represent higher adherence.


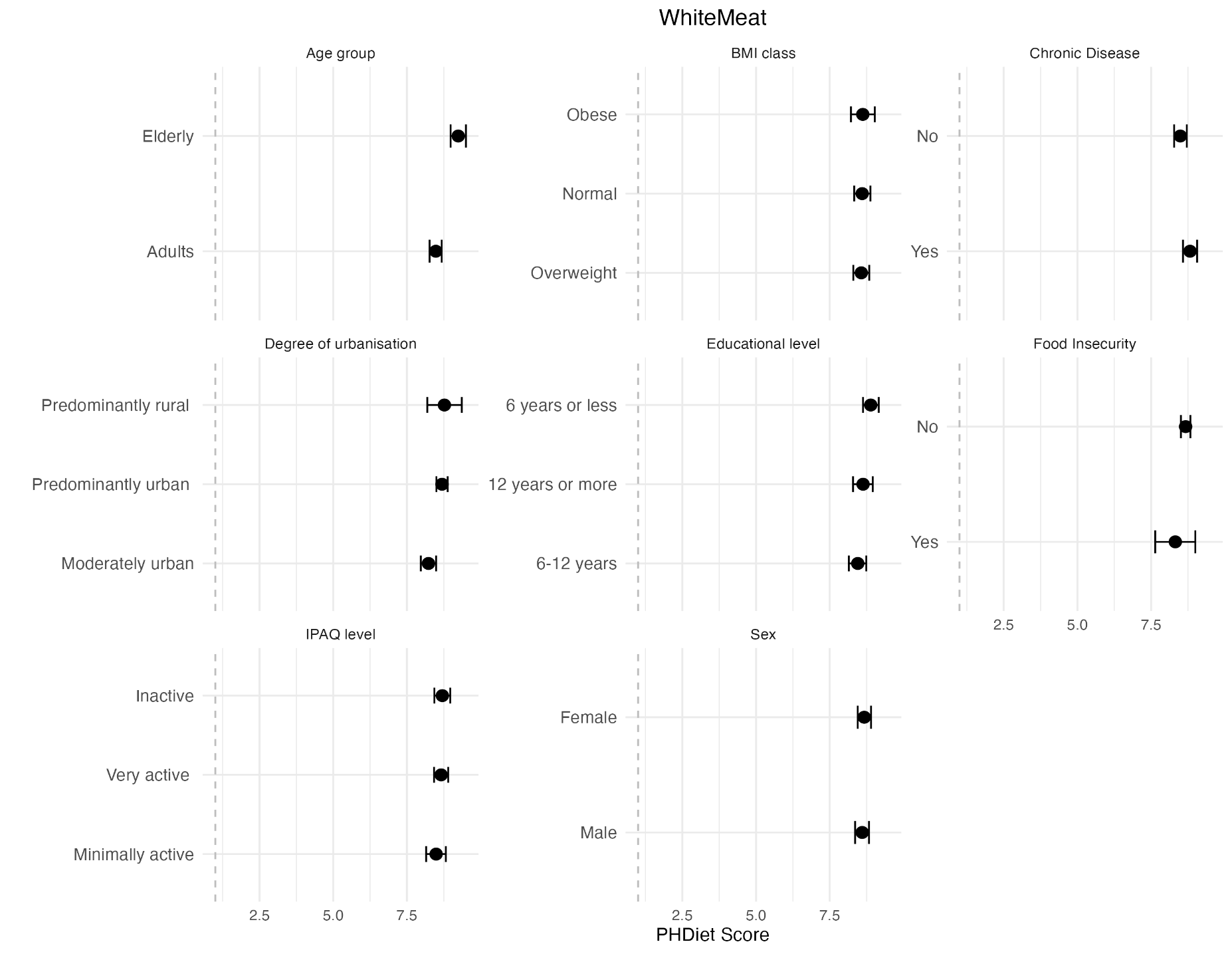


**Figure S1.8.** Average PHDiet Component Subscore – **White Meat** - per category of the sociodemographic and health-related variables under study. Higher scores represent higher adherence.


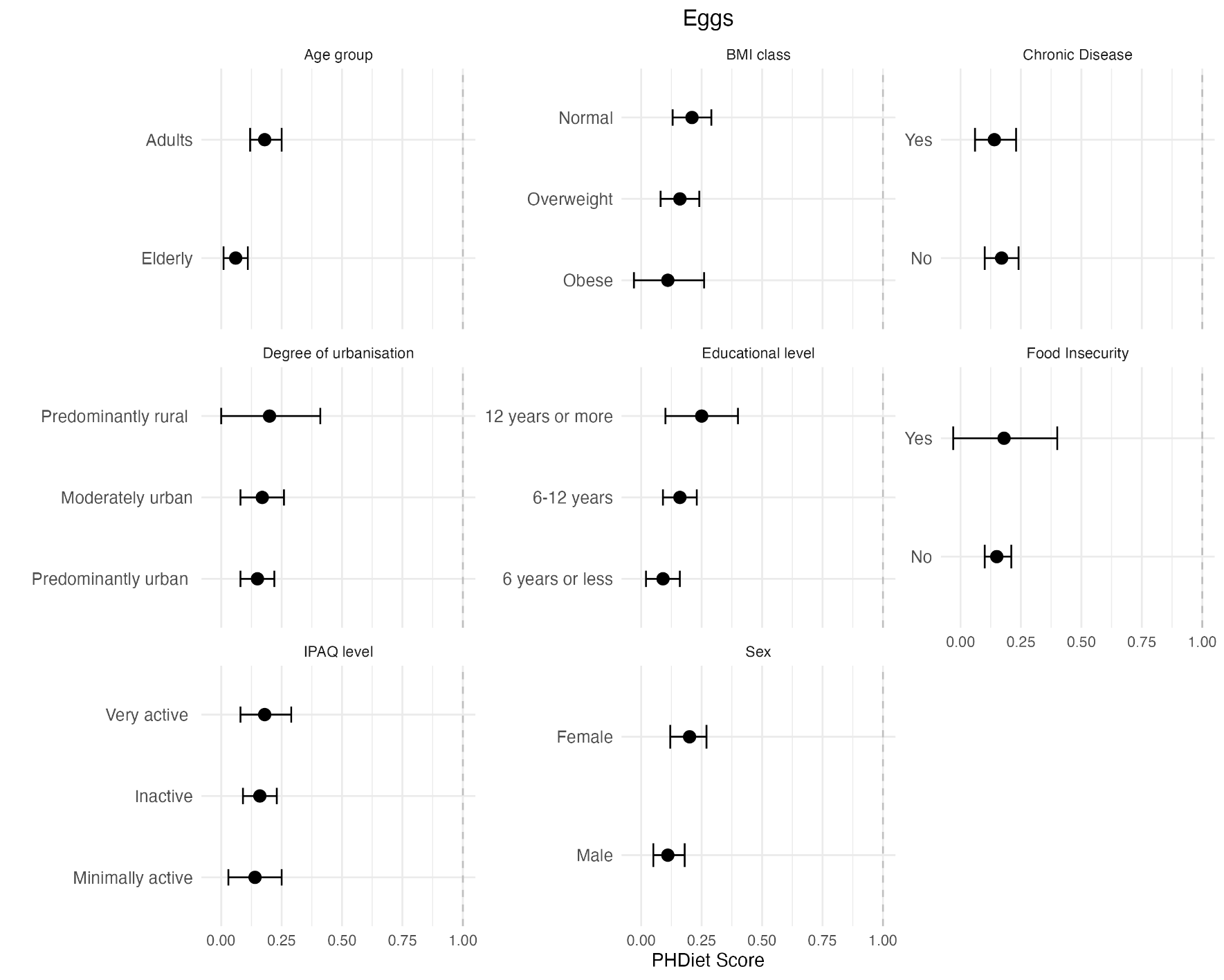


**Figure S1.9.** Average PHDiet Component Subscore – **Eggs** - per category of the sociodemographic and health-related variables under study. Higher scores represent higher adherence.


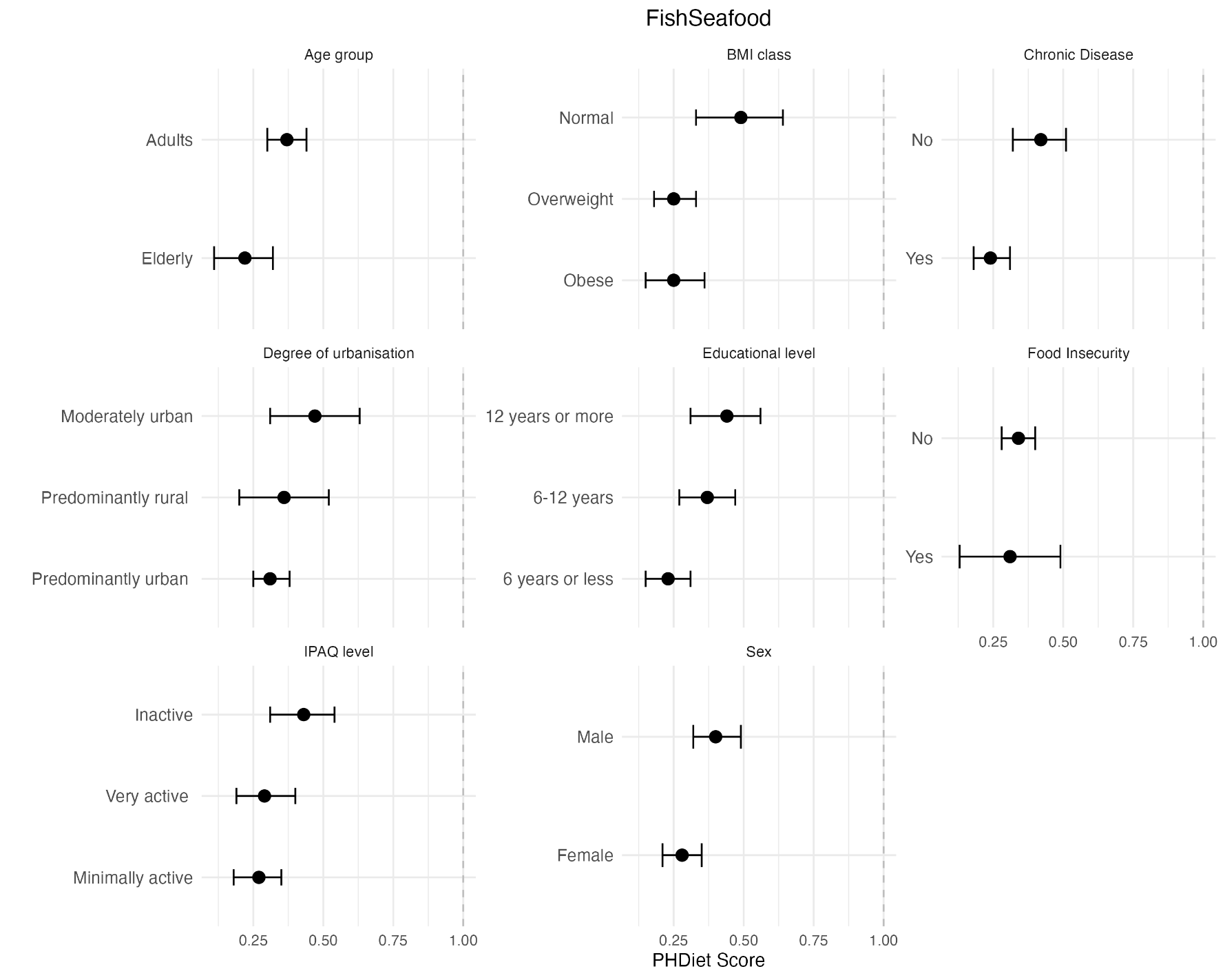


**Figure S1.10.** Average PHDiet Component Subscore – **Fish and Seafood** - per category of the sociodemographic and health-related variables under study. Higher scores represent higher adherence.


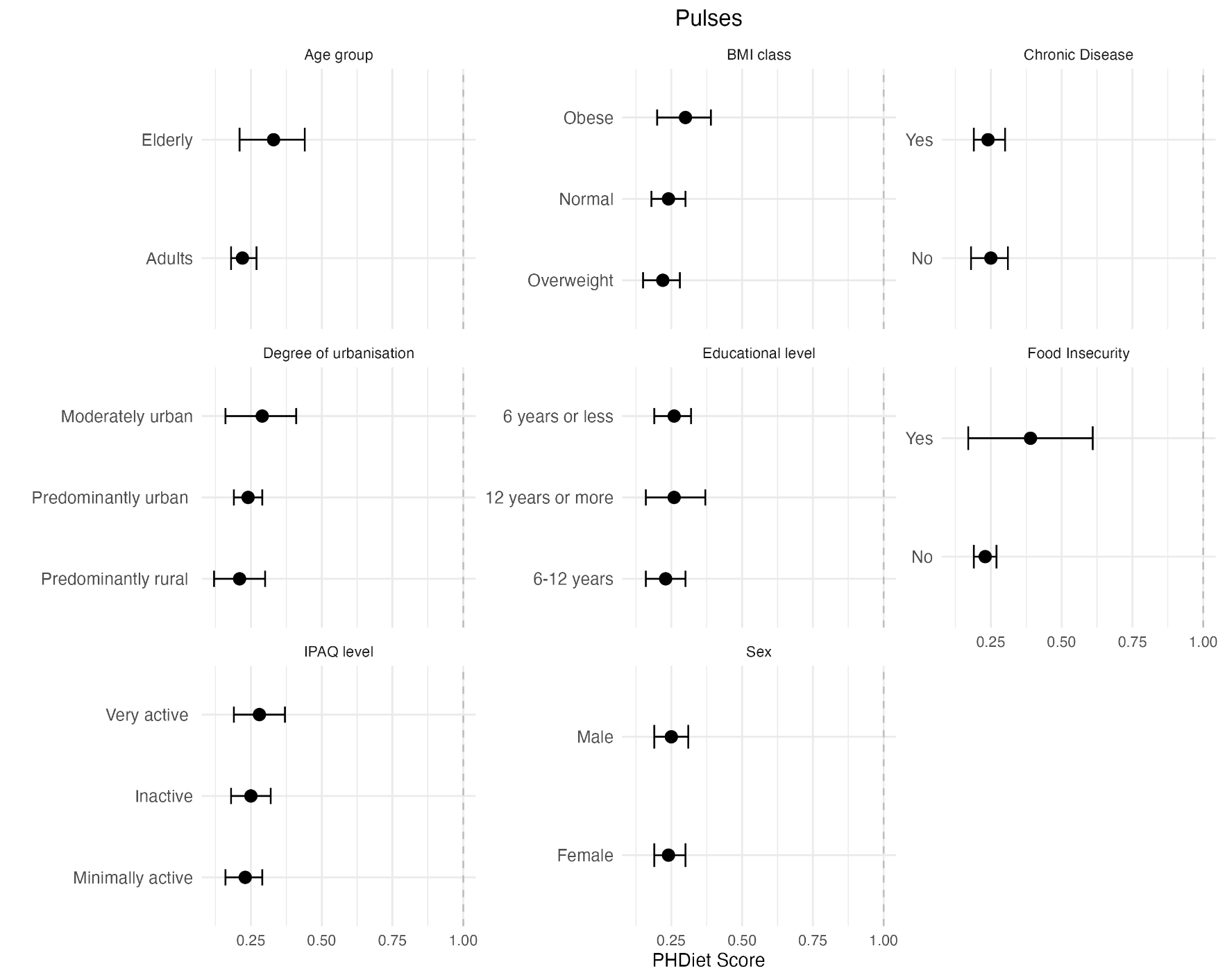


**Figure S1.11.** Average PHDiet Component Subscore – **Pulses** - per category of the sociodemographic and health-related variables under study. Higher scores represent higher adherence.


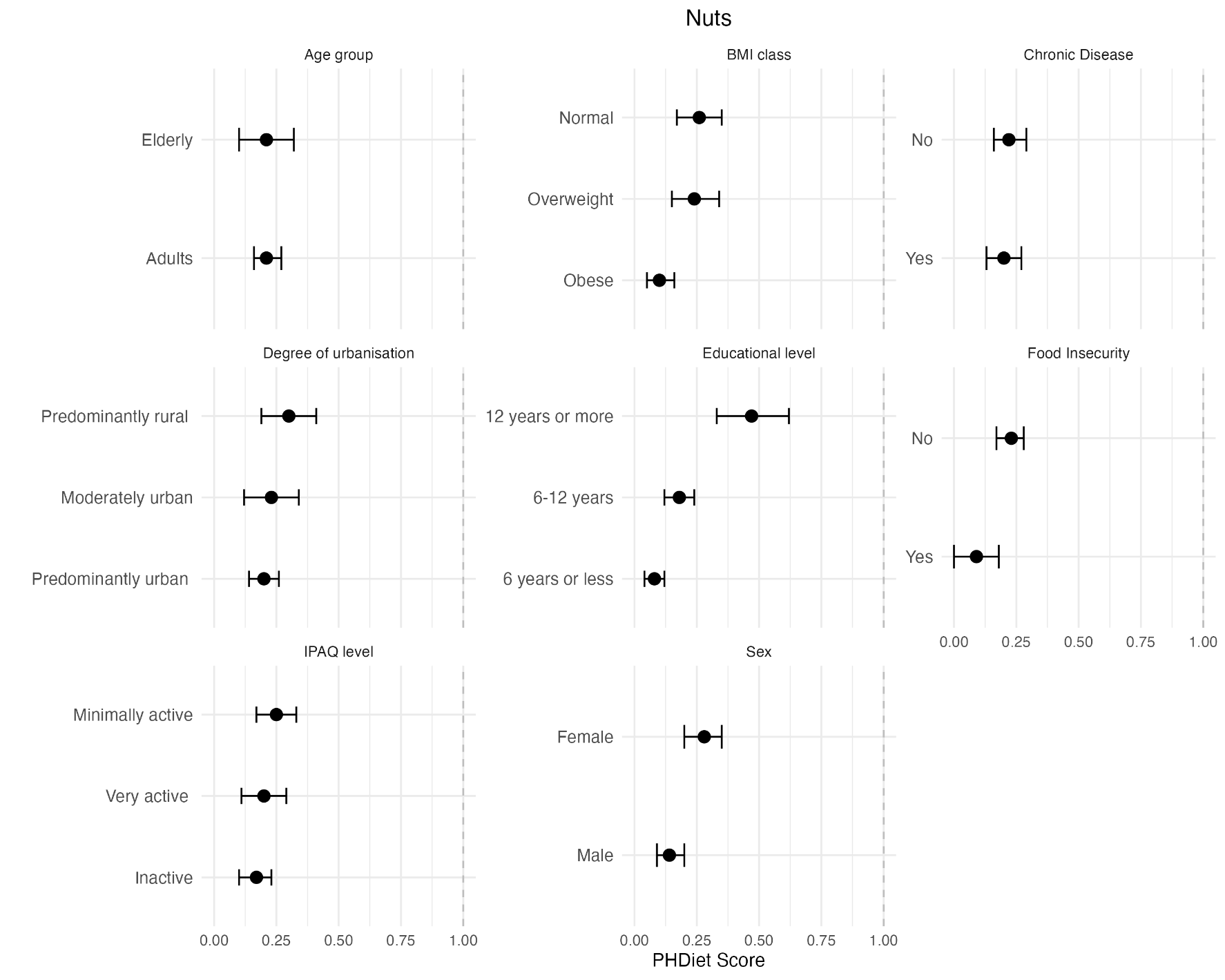


**Figure S1.12.** Average PHDiet Component Subscore – **Nuts** - per category of the sociodemographic and health-related variables under study. Higher scores represent higher adherence.


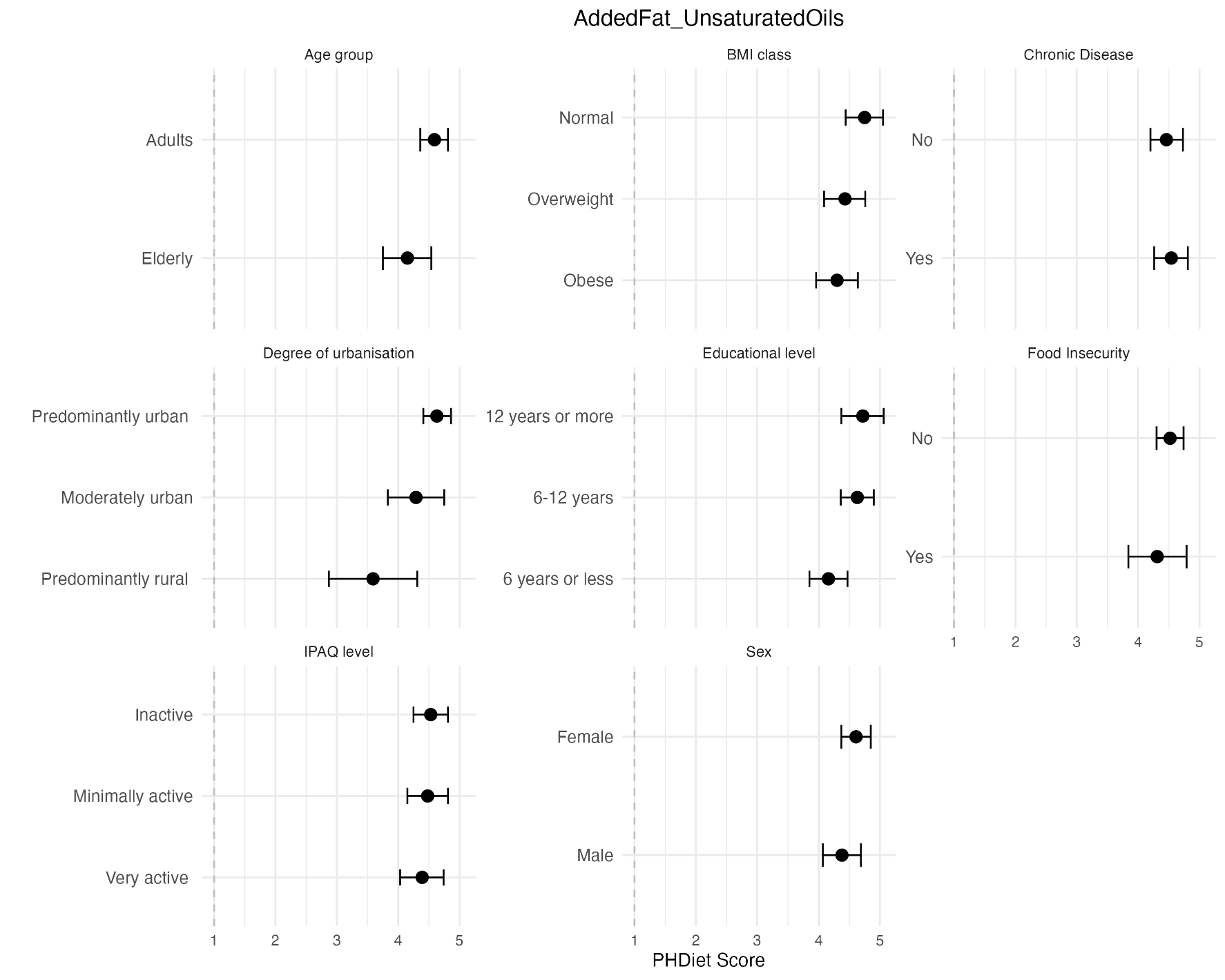


**Figure S1.13.** Average PHDiet Component Subscore – **Added fats – Unsaturated Oils** - per category of the sociodemographic and health-related variables under study. Higher scores represent higher adherence.


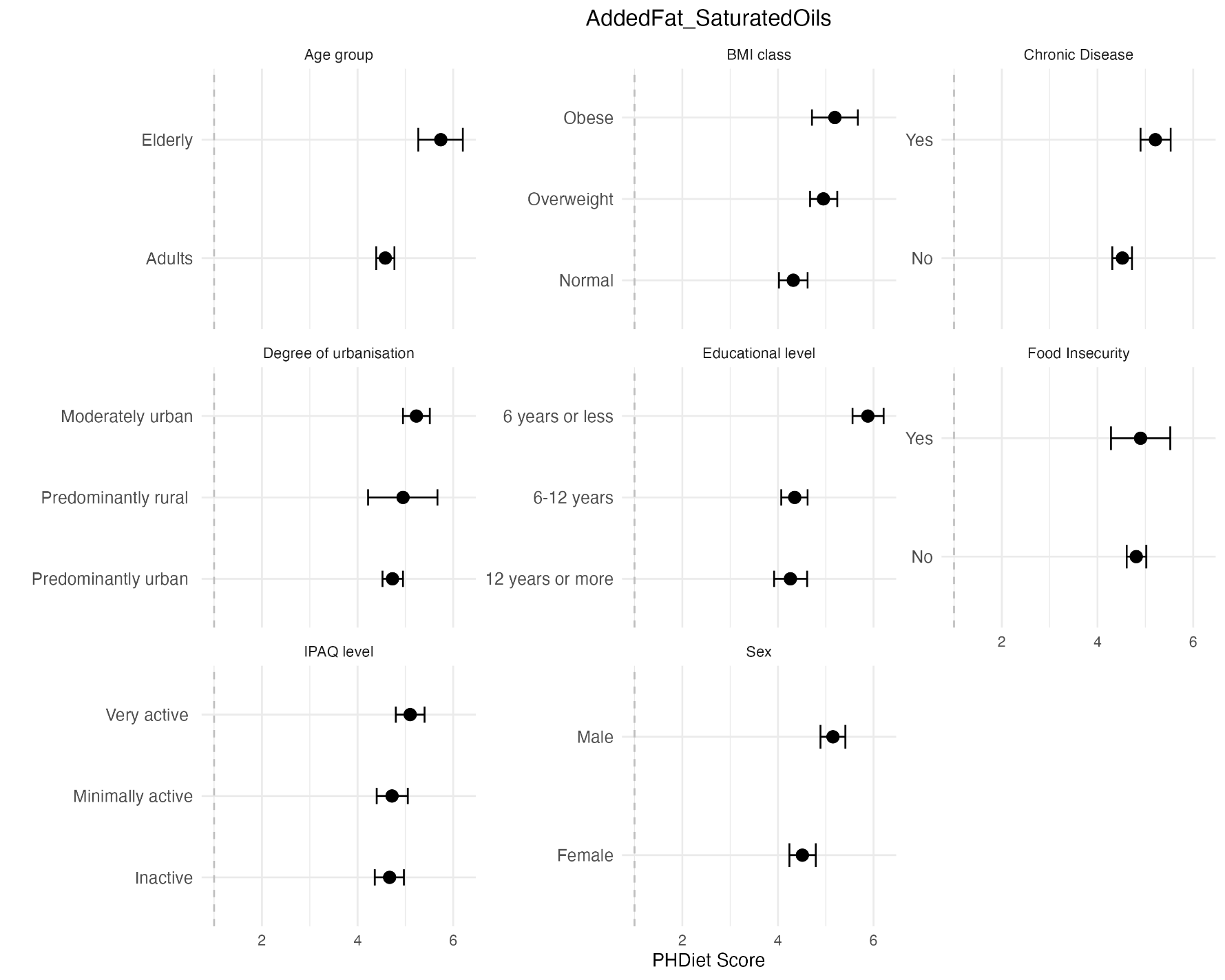


**Figure S1.14.** Average PHDiet Component Subscore – **Added Fat – Saturated oils** - per category of the sociodemographic and health-related variables under study. Higher scores represent higher adherence.


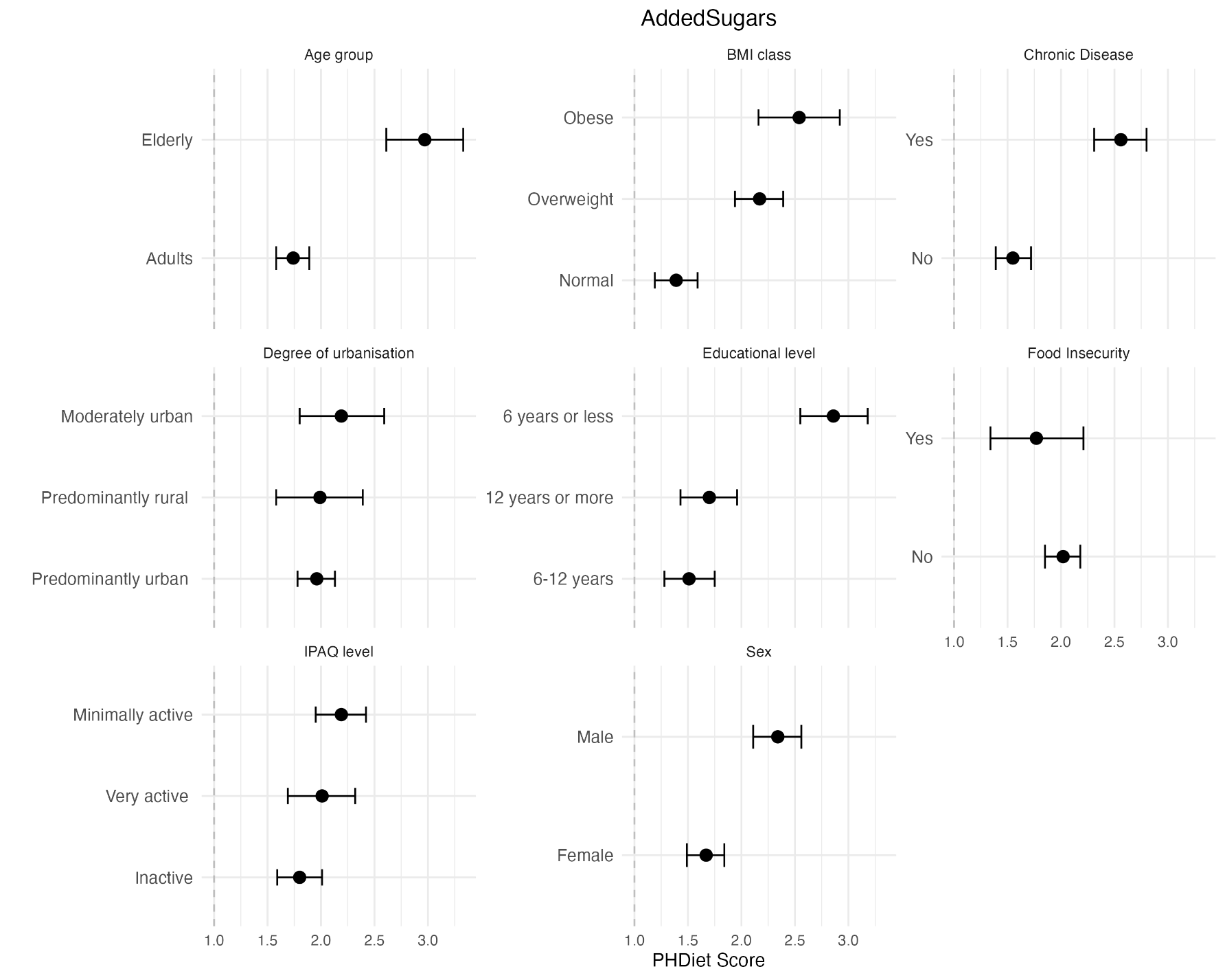


**Figure S1.15.** Average PHDiet Component Subscore – **Added Sugars** - per category of the sociodemographic and health-related variables under study. Higher scores represent higher adherence.
