# Supplementary material for: Adherence to the EAT-Lancet Planetary Health Diet in Portugal and its associations with socioeconomic and lifestyle factors
Source: Eur J Nutr. 2025 Apr 9;64(4):152. doi: 10.1007/s00394-025-03661-6 (PMC11982082; doi:10.1007/s00394-025-03661-6)
Supplement: Supplementary file 2 — Supplementary file2 (DOCX 454 KB) [file 394_2025_3661_MOESM2_ESM.docx]

**Supplementary material 2**

**Adherence to the Eat-Lancet-Planetary Health Diet in Portugal and its associations with socioeconomic and lifestyle factors**

Catarina Carvalho^1,2,3^, Daniela Correia^1,2,4^, Carla Lopes^1,2,4^, Duarte Torres^1,2,3^

^1^ EPIUnit – Instituto de Saúde Pública, Universidade do Porto, Porto, Portugal.

^2^ Laboratório para a Investigação Integrativa e Translacional em Saúde Populacional (ITR), Porto, Portugal

^3^ Faculdade de Ciências da Nutrição e Alimentação, Universidade do Porto, Porto, Portugal.

^4^ Departamento de Ciências da Saúde Pública e Forenses e Educação Médica, Faculdade de Medicina, Universidade do Porto, Porto, Portugal.

**Table of Contents – Supplementary tables**

[**Table S2.1.** Daily energy intake contributions (EC%) for the Planetary Health Diet-2 (PHDiet-2 **alternative version**) components and respective scoring system. **SENSITIVITY ANALYSIS** 2](#_Toc189583836)

[**Table S2.2.** Average Planetary Health Diet Score-2 (PHDiet-score-2 – **alternative version**) in the Portuguese population, the Portuguese National dietary survey (IAN-AF 2015-2016), n=3852. – **SENSITIVITY ANALYSIS** 3](#_Toc189583837)

[**Table S2.3.** Construct validity of Planetary Health Diet-2 (PHDiet-2, alternative version) score, based on PHDiet terciles (T1-T3) estimated for the Portuguese National dietary survey (IAN-AF 2015-2016), n=3852. **SENSITIVITY ANALYSIS** 4](#_Toc189583838)

**Table of Contents – Supplementary figures**

[**Figure S2.1.** Odds ratio for sociodemographic and health-related factors associations with PHDiet-score-2 (**alternative version**) tertiles in the Portuguese National Dietary Survey (IAN-AF 2015-2016), n = 3852.*. **SENSITIVITY ANALYSIS** 5](#_Toc189583842)

[**Figure S2.2.** Average nutrient intakes (in g/2500 kcal) by PHDiet-2 (**alternative version**) tercile (Low: T1; High: T3) in the Portuguese National Dietary Survey (IAN-AF 2015-2016), n = 3852. The average PHDiet score was 21.9 for T1 and 50.2 for T3. **SENSITIVITY ANALYSIS** 6](#_Toc189583843)

[**Figure S2.3.** Construct validity models applied to the different PHDiet-2 (alternative version) component subscores. Reference PHDiet tercile: “High PHDiet score” **–** The bars represent the odds of having a low PHDiet component subscores. HEI = Healthy Eating Index; AnimalProtein = Per 10 g animal protein; GHGE = per kg Greenhouse gas emissions; Land use = per m2/y Land use. **SENSITIVITY ANALYSIS** 7](#_Toc189583844)

**Table S2.1.** Daily energy intake contributions (EC%) for the Planetary Health Diet-2 (PHDiet-2 **alternative version**) components and respective scoring system. **SENSITIVITY ANALYSIS**

| **Dietary components** | **Scoring System** | **Planetary Health Diet** |
| --- | --- | --- |
|  |  | Energy contribution (EC%)^1^ |
|  |  | *mean*  *(recommended range)* |
| Whole grains | 0 < EC% < 60 🡪proportional score 0 - 10 | 32.4 (0.0-60%) |
| Tubers | EC% = 0 🡪 score: 0  EC% > 3.1 🡪 score: 0  0 < EC% ≤ 1.6 🡪 score: 10  1.6 ≤ EC% ≤ 3.1 🡪 proportional score 10 – 0 | 1.6 (0.0-3.1) |
| Vegetables | EC% < 2.1 🡪 score: 0  2.1 < EC% ≤ 6.2 🡪 proportional score 0 – 10  EC% > 6.2 🡪 score: 10 | 3.1 (2.1-6.2) |
| Fruit | EC% < 2.5 🡪 score: 0  2.5 < EC% ≤ 7.6 🡪 proportional score 0 – 10  EC% > 7.6 🡪 score: 10 | 5.0 (2.5-7.6) |
| Dairy | EC% = 0 🡪 score: 0  EC% > 12.2 🡪 score: 0  0 < EC% ≤ 6.1 🡪 score: 10  6.1 ≤ EC% ≤ 12.21 proportional score 10– 0 | 6.1 (0.0-12-2) |
| Red Meat | EC% = 0 🡪 score: 10  0 < EC% ≤ 2.4 🡪 proportional score 10 – 0  EC% > 2.4 🡪 score: 0 | 1.2 (0.0-2.4) |
| White Meat | EC% = 0 🡪 score: 10  0 < EC% ≤ 5.0 🡪 proportional score 10 – 0  EC% > 5.0 🡪 score: 0 | 2.5 (0.0-5.0) |
| Eggs | EC% = 0 🡪 score: 0  EC% > 1.5 🡪 score: 0  0 < EC ≤ 0.8🡪 score: 10  0.8 ≤ EC% ≤ 1.5 🡪 proportional score 10 – 0 | 0.8 (0.0-1.5) |
| Fish and Seafood | EC% = 0 🡪 score: 0  EC% > 5.7 🡪 score: 0  0 < EC% ≤ 1.6🡪 score: 10  1.6 ≤ EC% ≤ 5.7 🡪 proportional score 10 – 0 | 1.6 (0.0-5.7) |
| Pulses | EC% = 0 🡪 score: 0  0 < EC% ≤ 15.1 🡪 proportional score 0 – 10  EC% > 15.1 🡪 score: 10 | 11.3 (0.0-15.1) |
| Nuts | EC% = 0 🡪 score: 0  0 < EC% ≤ 17.5 🡪 proportional score 0 – 10  EC% > 17.5 🡪 score: 10 | 11.6 (0.0-17.5) |
| Added Fat – Unsaturated Oils | EC% < 7.1 🡪 score: 0  EC% > 28.3 🡪 score: 0  7.1 < EC% ≤ 14.1🡪 score: 10  14.1 ≤ EC% ≤ 28.3 🡪 proportional score 10 – 0 | 14.1 (7.1-28.3) |
| Added Fat – Saturated Oils | 0 < EC% ≤ 3.8 🡪 proportional score 10 – 0  EC% > 3.8🡪 score: 0 | 3.8 (0.0-3.8) |
| Added Sugar | 0 < EC% ≤ 4.8 🡪 proportional score 10 – 0  EC %> 4.8🡪 score: 0 | 4.8 (0.0-4.8) |
| ^1.^ Calculated from the scientific targets for a planetary health diet proposed by Willet et al. (2019) [5]; | | |

**Table S2.2.** Average Planetary Health Diet Score-2 (PHDiet-score-2 – **alternative version**) in the Portuguese population, the Portuguese National dietary survey (IAN-AF 2015-2016), n=3852. – **SENSITIVITY ANALYSIS**

|  | **Average**  **PHDiet-score** |
| --- | --- |
|  |  |
|  | Mean (95%CI) |
| Overall | 61.5 (60.7; 62.2) |
| Sex |  |
| Female | 62.7 (61.7; 63.7) |
| Male | 60.2 (59.1; 61.2) |
| Age group |  |
| Adults (18-64 years) | 60.1 (59.3; 61.0) |
| Elderly (≥65 years) | 66.5 (65.0; 68.0) |
| Educational level |  |
| ≤ 6 years | 64.8 (63.7; 65.9) |
| 6-12 years | 59.4 (58.4; 60.5) |
| >12 years | 60.7 (59.3; 62.2) |
| Degree of urbanisation |  |
| Predominantly urban | 61.8 (60.9; 62.7) |
| Moderately urban | 59.9 (58.3; 61.5) |
| Predominantly rural | 60.9 (59.2; 62.6) |
| Food Insecurity |  |
| No | 61.5 (60.7; 62.4) |
| Yes | 60.7 (58.8; 62.7) |
| BMI class |  |
| Normal | 59.3 (58.0; 60.7) |
| Overweight | 62.6 (61.3; 63.9) |
| Obese | 62.4 (60.9; 63.9) |
| Chronic Disease |  |
| No | 59.5 (58.6; 60.4) |
| Yes | 64.0 (62.8; 65.1) |
| IPAQ level |  |
| Inactive | 60.7 (59.5; 61.8) |
| Minimally active | 61.5 (60.2; 62.8) |
| Very active | 62.2 (60.8; 63.7) |

**Table S2.3.** Construct validity of Planetary Health Diet-2 (PHDiet-2, alternative version) score, based on PHDiet terciles (T1-T3) estimated for the Portuguese National dietary survey (IAN-AF 2015-2016), n=3852. **SENSITIVITY ANALYSIS**

| **Parameters** | **Hypothesis^1^** | **Low (T1) vs High (T2) PHDiet-score-2** | | **Intermediate (T2) vs High (T3) PHDiet-score-2** | |
| --- | --- | --- | --- | --- | --- |
|  |  | OR^2^ | 95%CI | OR^2^ | 95%CI |
| Healthy eating index (HEI) | *Increasing HEI leads to lower odds of having lower PHDiet adherence (T1 vs T3),* | **0.86** | **0.84; 0.89** | **0.93** | **0.90; 0.95** |
| Environmental impact – Land Use (m^2^/year) | *Increasing dietary environmental impact (LU and GHGE) leads to higher odds of having lower PHDiet adherence (T1 vs T3),* | **1.18** | **1.15; 1.21** | **1.12** | **1.09; 1.15** |
| Environmental impact – GHGE (kgCO_2_eq/day) |  | **1.34** | **1.28; 1.39** | **1.20** | **1.15; 1.25** |
| Animal protein (per each 10g/day) | *Increasing animal protein intake leads to higher odds of having lower PHDiet adherence (T1 vs T3),* | **1.25** | **1.19; 1.31** | **1.13** | **1.07; 1.18** |
| ^1^ According to the rationale of Willet et al. (2019) [5].  ^2^Adjusted for sex and age group. Reference category: High PHDiet score-2 | | | | | |

**Figure S2.1.** Odds ratio for sociodemographic and health-related factors associations with PHDiet-score-2 (**alternative version**) tertiles in the Portuguese National Dietary Survey (IAN-AF 2015-2016), n = 3852.*. **SENSITIVITY ANALYSIS**

*Multinomial logistic regression models adjusted for sex, age and educational level. The error bars indicate the 95%CI.

Reference PHDiet tercile: “High PHDiet score. Variables reference categories – Sex: Female; Age group: Adults; Educational level: >12 years; Degree of urbanisation: Predominantly urban; Food insecurity: No; BMI class: Normal weight; Chronic disease: No; IPAQ level: Inactive.

**Figure S2.2.** Average nutrient intakes (in g/2500 kcal) by PHDiet-2 (**alternative version**) tercile (Low: T1; High: T3) in the Portuguese National Dietary Survey (IAN-AF 2015-2016), n = 3852. The average PHDiet score was 21.9 for T1 and 50.2 for T3. **SENSITIVITY ANALYSIS**

* Significant differences between High and Low terciles (two-sample t-test, p-value < 0.05)


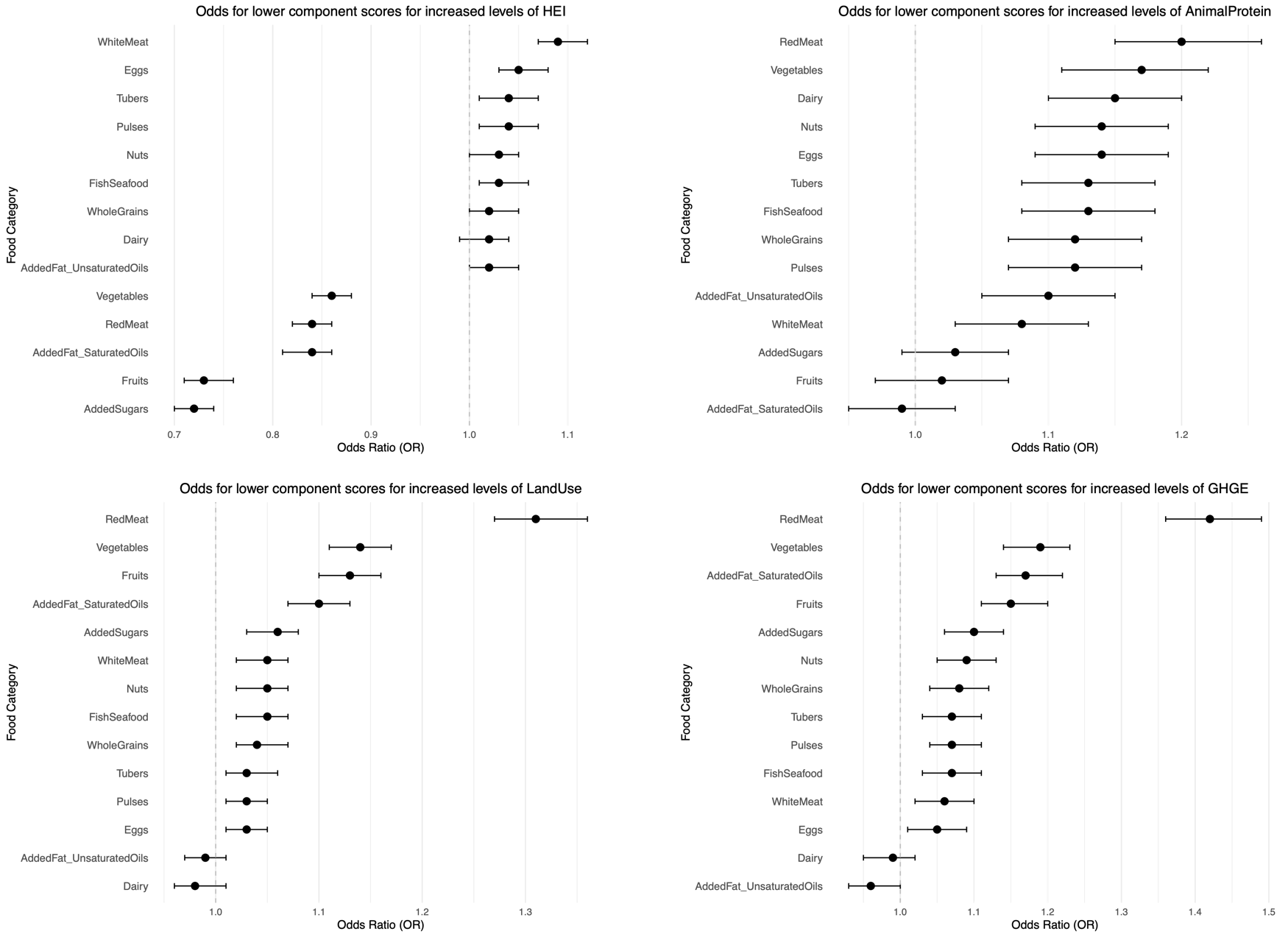


**Figure S2.3.** Construct validity models applied to the different PHDiet-2 (alternative version) component subscores. Reference PHDiet tercile: “High PHDiet score” **–** The bars represent the odds of having a low PHDiet component subscores. HEI = Healthy Eating Index; AnimalProtein = Per 10 g animal protein; GHGE = per kg Greenhouse gas emissions; Land use = per m2/y Land use. **SENSITIVITY ANALYSIS**
